# Patients experiencing statin-induced myalgia exhibit a unique program of skeletal muscle gene expression following statin re-challenge

**DOI:** 10.1371/journal.pone.0181308

**Published:** 2017-08-03

**Authors:** Marshall B. Elam, Gipsy Majumdar, Khyobeni Mozhui, Ivan C. Gerling, Santiago R. Vera, Hannah Fish-Trotter, Robert W. Williams, Richard D. Childress, Rajendra Raghow

**Affiliations:** 1 Department of Veterans Affairs Medical Center-Memphis, Memphis, Tennessee, United States of America; 2 Department of Pharmacology, University of Tennessee Health Sciences Center, Memphis, Tennessee, United States of America; 3 Department of Medicine, University of Tennessee Health Sciences Center, Memphis, Tennessee, United States of America; 4 Department of Preventive Medicine, University of Tennessee Health Sciences Center, Memphis, Tennessee, United States of America; 5 Department of Genetics, Genomics and Informatics, College of Medicine, University of Tennessee Health Science Center, Memphis, Tennessee, United States of America; University of Valencia, SPAIN

## Abstract

Statins, the 3-hydroxy-3-methyl-glutaryl (HMG)-CoA reductase inhibitors, are widely prescribed for treatment of hypercholesterolemia. Although statins are generally well tolerated, up to ten percent of statin-treated patients experience myalgia symptoms, defined as muscle pain without elevated creatinine phosphokinase (CPK) levels. Myalgia is the most frequent reason for discontinuation of statin therapy. The mechanisms underlying statin myalgia are not clearly understood. To elucidate changes in gene expression associated with statin myalgia, we compared profiles of gene expression in skeletal muscle biopsies from patients with statin myalgia who were undergoing statin re-challenge (cases) *versus* those of statin-tolerant controls. A robust separation of case and control cohorts was revealed by Principal Component Analysis of differentially expressed genes (DEGs). To identify putative gene expression and metabolic pathways that may be perturbed in skeletal muscles of patients with statin myalgia, we subjected DEGs to Ingenuity Pathways (IPA) and DAVID (Database for Annotation, Visualization and Integrated Discovery) analyses. The most prominent pathways altered by statins included cellular stress, apoptosis, cell senescence and DNA repair (TP53, BARD1, Mre11 and RAD51); activation of pro-inflammatory immune response (CXCL12, CST5, POU2F1); protein catabolism, cholesterol biosynthesis, protein prenylation and RAS-GTPase activation (FDFT1, LSS, TP53, UBD, ATF2, H-ras). Based on these data we tentatively conclude that persistent myalgia in response to statins may emanate from cellular stress underpinned by mechanisms of post-inflammatory repair and regeneration. We also posit that this subset of individuals is genetically predisposed to eliciting altered statin metabolism and/or increased end-organ susceptibility that lead to a range of statin-induced myopathies. This mechanistic scenario is further bolstered by the discovery that a number of single nucleotide polymorphisms (e.g., *SLCO1B1*, *SLCO2B1* and *RYR2)* associated with statin myalgia and myositis were observed with increased frequency among patients with statin myalgia.

## Introduction

Large-scale clinical trials conducted over the past two decades have firmly established statins, the inhibitors of the rate-limiting enzyme of cholesterol biosynthesis, HMG-CoA reductase, as highly effective in the prevention of atherosclerotic cardiovascular disease (ASCVD). Current guidelines recommend statins as a first-line drug therapy for lowering LDL-cholesterol in patients at risk of ASCVD [1]. Statins effectively reduce plasma levels of LDL cholesterol up to 60% by inhibiting cholesterol biosynthesis in the liver. However, statin treatment can also result in muscle-related adverse effects ranging in severity from mild to moderate muscle pain (myalgia) to severe and even life-threatening myopathies (e.g., myositis and rhabdomyolysis) [2]. Although the occurrence of the more severe forms of myopathy with statin therapy is rare, myalgia defined as muscle pain in the absence of increased creatine phosphokinase (CPK) occurs in up to 10% of statin treated patients and frequently results in discontinuation of statin therapy [3–5]. Patients experiencing statin myalgia report symptoms that interfere with their normal daily activity with over half totally precluding moderate exertion [6]. Inability to tolerate statin due to myalgia has emerged as a significant barrier to effective control of LDL cholesterol, thereby exposing these individuals to increased risk of cardiovascular disease [6, 7]. Whereas some risk factors for the rare, more serious syndromes such as myositis and rhabdomyolysis have been identified, information regarding the underlying pathogenesis and predisposing factors for statin-induced myalgia remains limited [2, 3, 8]. Conventional risk factors for myositis are noticeably absent in the majority of cases of statin-related myalgia [7]. These observations suggest the presence of an underlying, perhaps genetic susceptibility in patients with statin myalgia that elicits a unique pathophysiological response to statins. To gain insight into the cellular and molecular mechanisms of statin-induced myalgia, we performed gene expression analysis on muscle biopsy specimens obtained following statin re-challenge in patients with previous history of statin myalgia. Our analyses have revealed that a number of gene regulatory pathways that impinge on the structural integrity and performance of the skeletal muscle and its response to post-inflammatory repair and regeneration are altered by statins.

## Materials and methods

### Ethics statement and study subjects

Prior to initiation of the study, the study protocol was reviewed and approved by the Investigational Review Boards (IRB) of the Veteran's Affairs Medical Center (VAMC) Memphis and University of Tennessee Health Sciences Center, Memphis. Prior to participation, all study participants provided written informed consent using IRB approved procedures and consent documents. Patients referred to the VAMC Lipid Clinic, who met the diagnostic criteria for statin myalgia, participated in this study. Eligible cases had a history of discontinuation of one or more statins due to muscle symptoms without significant elevation in CPK (>5-fold above normal). Statin myalgia was assessed using the Naranjo probability score [9]. All cases had experienced reversible symptoms of myalgia that recurred following re-challenge with one or more statin(s). Asymptomatic patients who adhered to statin therapy for at least 6 months prior to evaluation were defined as statin-tolerant controls. Patients with advanced renal disease (GFR < 30 ml/min), liver disease (active hepatitis and/or serum transaminase >3-fold above normal), and HIV and/or current treatment with protease inhibitors were excluded. Patients with recent history (within one year) of acute vascular syndrome (unstable angina or myocardial infarction; stroke or transient ischemic attack) or current symptoms of angina pectoris as well as patients with a history of bleeding disorder and those prescribed either oral anticoagulant or thienopyridine antiplatelet medication were also excluded. In order to avoid potential confounding effects of race and sex on muscle gene expression in this relatively limited sample of patients only male subjects of European descent were enrolled in this study.

Patients with a history of statin myalgia (hereafter referred to as cases) underwent re-challenge with simvastatin 20 mg per day or an alternative statin as per lipid clinic protocol. Cases were evaluated after 2 weeks and the statin dose was increased if tolerated. Statin tolerant participants (controls) continued statin therapy as prescribed by their clinical care provider. All participants continued statin therapy for a total of 8 weeks or until muscle symptoms required discontinuation, at which time muscle biopsies were performed. A complete medical history was recorded including details of prior statin tolerance or intolerance, concomitant medications, and co-morbid diseases. At the final study visit blood samples were obtained for laboratory tests and for isolation of peripheral blood mononuclear cells (PBMCs) and muscle biopsies. A total of 26 subjects underwent muscle biopsy (16 cases and 10 controls).

### Collection of blood and muscle specimens

From individual patients, 7–8 ml of blood was collected in EDTA-containing tubes. Whole blood was passed through LeukoLOCK filters using an evacuated tube as vacuum source and the leukocytes were captured on the filter. The filter was flushed once with 3 ml of PBS to eliminate trapped red blood cells and then flushed again with 3 ml of RNA*later* to stabilize the RNA in the filter-bound leukocytes; filters were stored at –80°C until used for extraction of nucleic acids. Muscle biopsies were taken from the lateral portion of the quadriceps femoris muscle under local anesthetic (xylocaine), using Bergstrom needles (Cadence Science, Cranston R.I.) as described by Mandarino et al. [10]. To reduce the risk of bleeding, participants were instructed to avoid aspirin or aspirin containing medications as well as non-steroidal anti-inflammatory medications for at least 5 days prior to biopsy procedure. Muscle specimens were snap-frozen in liquid nitrogen and stored at –80°C until used for extraction of mRNA and DNA [11].

### Extraction of RNA and DNA

The method used to co-extract RNA and DNA from PBMCs and skeletal muscle has been described in detail [12]. Total RNA was extracted from PBMCs using the LeukoLOCK Total RNA Isolation system (Ambion, Life Technologies, CA). Filters containing PBMCs, stored at –80°C, were allowed to thaw at room temperature and the RNAlater was expelled with a 3 ml syringe. The filter-bound PBMCs were lysed with 4 ml TRIzol reagent and RNA was extracted. Samples were filtered through the spin cartridges, washed and RNA was eluted with water at room temperature. The eluted RNA was stored at –80°C. Total RNA was also extracted from muscle by the TRIzol method. The genomic DNA was extracted from muscle (10 mg) by using the QIAamp MinElute Column (Qiagen, CA). Tissues were digested in the presence of proteinase K at 56°C overnight. The entire lysate was applied to the QIAamp MinElute column, filtered and washed by centrifugation and then DNA was eluted with 1 mM Tris-EDTA, pH 8.0 [12].

### Gene expression profiling

The yield and integrity of RNAs were assessed by measuring A_260_/A_280_ ratios in a spectrophotometer and by size-fractionation in 1% agarose gels, respectively. Two hundred ng aliquots of total RNA per sample were used for cDNA and cRNA synthesis using Illumina TotalPrep RNA Amplification Kit (Applied Biosystems/Ambion, Austin, TX). Aliquots of amplified and labeled cRNA (750–1500 ng) were hybridized to Illumina HT-12 V4 Expression BeadChips containing probes to evaluate expression levels of ~47,231 transcripts (Illumina Inc., San Diego, CA). Probe level expression signals were loaded to Illumina BeadStudio GX Module software (version 3.4.0) to generate an output file. This was loaded to GeneSpring (version 7.3.1, Silicon Genetics, Redwood, CA) for quality check and data normalization as previously described [13]. One sample had low hybridization signal and was dropped. Unsupervised hierarchical clustering identified two outlier samples that were also excluded. For probe quality control, the 47,231 probes from the 23 remaining samples were filtered to identify 21,344 that had a positive expression flag of > 0.99 in at least three samples. Gene expression ratios for individual genes corrected for vitamin D level and chip batch (see below) are available in Supplemental Table 1. The data discussed in this publication have been deposited in NCBI's Gene Expression Omnibus (Edgar *et al*., 2002) and are accessible through GEO Series accession number GSE97254 (https://www.ncbi.nlm.nih.gov/geo/query/acc.cgi?acc=GSE97254).

**Table 1:** Demographic and laboratory characteristics of cases and controls ^§^.

Characteristic		CASES (N = 16)	CONTROLS (N = 10)	*Nominal P Value
**Age years/(Range)**		**65.4 ± 1.5 (59–72)**	**60.9 ± 2.6 (46–78)**	**0.08**
**Body Mass Index (BMI)**		**30.5 ± 1.4**	**35.1 ± 1.9**	**0.97**
**Cardiovascular Disease**		**80.0%**	**60.0%**	**0.23**
**Hypertension**		**75.0%**	**50.0%**	**0.19**
**Coronary Artery Disease**		**43.8%**	**30.0%**	**0.48**
**Type II Diabetes**		**43.8%**	**40.0%**	**0.85**
**Musculoskeletal Disease**		**68.75%**	**40.0%**	**0.15**
Psychiatric Illness**		**37.5%**	**40.0%**	**0.90**
Depression		**25.0%**	**20%**	**0.77**
Anxiety Disorder		**12.5%**	**10.0%**	**0.85**
Posttraumatic Stress Disorder		**12.5%**	**10.0%**	**0.85**
**Thyroid Disease**		**6.25%**	**10.0%**	**0.73**
**Family History of Statin Intolerance**		**25.0%**	**0.0%**	**0.23**
**MEDICATIONS**				
Calcium Channel Blocker		**31.3%**	**40.0%**	**0.65**
ACEI/ARB		**56.3%**	**40.0%**	**0.42**
Beta Blocker		**37.5%**	**20.0%**	**0.35**
Diuretic		**37.5%**	**20.0%**	**0.35**
Fibrate		**25.0%**	**20.0%**	**0.77**
Ezetimibe		**12.5%**	**10.0%**	**0.85**
Niacin		**31.3%**	**20.0%**	**0.53**
Fish Oil		**56.3%**	**30.0%**	**0.19**
Nonsteroidal Antiinflammatory Drug (NSAID)		**18.8%**	**30.0%**	**0.51**
Serotonin Uptake Inhibitors (SSRI)		**25.0%**	**20.0%**	**0.77**
Metformin		**37.5%**	**30.0%**	**0.70**
Glitazone		**6.3%**	**0.0%**	**0.42**
Sulfonylurea		**18.8%**	**10.0%**	**0.55**
Insulin		**12.5%**	**10.0%**	**0.85**
Proton Pump Inhibitor (PPI)		**50.0%**	**30.0%**	**0.32**
Aspirin		**75.0%**	**50.0%**	**0.19**
Vitamin D		**50.0%**	**10.0%**	0.04*
Testosterone		**25.0%**	**10.0%**	**0.09**
**PRIOR STATIN MYALGIA WITH:**				
Simvastatin		**15 (93.8%)**	—-	
Atorvastatin		**6 (37.5%)**	—-	
Rosuvastatin		**10 (62.5%)**	—-	
Pravastatin		**6 (37.5%)**	—-	
Fluvastatin		**2 (12.5%)**	—-	
Lovastatin		**1 (6.25%)**	—-	
**Number of Statins associated with myalgia per patient** **(average and range)**		**2.5 (1–4)**	—-	
**Intensity of Statin Treatment associated with myalgia**				
High		6	—-	
Moderate		18	—-	
Low		9	—-	
**Symptoms Reported**				
Weakness		7 (46.7%)	—-	
Pain		11 (73.3%)	—-	
Cramps		8 (53.3%)	—-	
**Location of Symptoms**			—-	
Legs		13 (86.7%)	—-	
Shoulder/Arm		5 (33.3%)	—-	
Neck/Back		5 (33.3%)	—-	
**Bilateral symptoms**		15 (100%)	—-	
**Symptom Severity^&^**				
Mild		0 (0%)	—	
Moderate		3 (20%)	—	
Severe		12 (80%)	—	
**LABORATORY VALUES ^@^**	**(Normal Range)**			
Potassium	(3.5–5.0 mmol/L)	4.3 ± 0.1	4.1 ± 0.1	0.10
Calcium	(8.5–10.2 mg/dl)	9.4 ± 0.1	9.6 ± 0.1	0.91
Creatinine	(0.7–1.6 mg/dl)	0.98 ± 0.06	0.96 ± 0.06	0.43
CPK	(20–320 U/L)	115.5 ± 16.0	108.9 ± 0.4	0.38
Antinuclear Antigen (ANA) Positive		12.5%	20.0%	0.36
Sedimentation Rate	(0–20 mm HR)	13.8 ± 2.9	10.0 ± 1.3	0.14
Rheumatoid Factor (elevated)		7.7%	10.0%	0.93
Testosterone	(total) (241–827 ng/dl)	299 ± 32	200 ± 18	0.01*
Testosterone (free)	(7.2–24.0 pg/ml)	8.1 ± 0.9	6.9 ± 1.2	0.21
TSH	(0.4–4.7 uIU/ml)	1.88 ± 0.25	2.26± 0.34	0.81
Free T4	(0.75–2.45 ng/dL)	1.07 ± 0.05	1.08 ± 0.09	0.52
25-OH Vitamin D	(35–1a35 ng/ml)	30.7 ± 2.2	20.8 ± 3.3	0.01*
Baseline 25-OH Vitamin D ^#^	(35–135 ng/ml)	22.5 ± 1.8	20.2 ± 3.7	0.29
High Sensitivity C-Reactive Protein	(1.0–3.0 mg/L)	1.7 ± 0.4	0.2 ± 0.1	0.002*
Cholesterol (mg/dl)		214 ± 9	174 ± 9	0.002*
LDL-Cholesterol (mg/dl)		126 ± 10	90 ± 10	0.008*
HDL-Cholesterol (mg/dl)		43.9 ± 2.4	40.6 ± 2.7	0.19
Triglyceride (mg/dl)		224 ± 27	216 ± 43	0.44
			
**Statin used for re-challenge**				
Simvastatin		81.25%	80.0%	0.52
Atorvastatin		12.5%	10.0%	
Rosuvastatin		6.25%	10.0%	
**Re-challenge Statin Intensity**				
Low		6.25%	10.0%	0.88
Moderate		87.5%	80.0%	
High		6.25%	10.0%	
**Symptoms reported during re-challenge**		75.0%	—-	
			

^**§**^ Data are mean ± sem of clinical characteristics of cases vs controls.

*Nominal P values calculated using Student’s T-Test for continous variables and Chi Square Analysis (Pearson R) for discrete variables.

** composite of depression, anxiety disorder and PTSD.

^**@**^ Unless otherwise noted characteristics represent those present at the time of statin re-challenge and muscle biopsy. Baseline vitamin D is value prior to vitamin D supplementation.

^&^ Symptom severity: Mild = symptoms present but easily ignored, Moderate = symptoms cannot be ignored but do not interfere with daily activities; Severe = symptoms interfere with daily activity.

^#^ Vitamin D level prior to supplementation.

### Data analysis and statistics

We performed principal component analysis on the filtered expression data and this identified three additional outlier samples that were also excluded. PC analysis on the remaining 20 samples (13 cases and 7 controls) was then done to derive the major PCs. The first 3 PCs explain over 98.5% of the total variance and we used these to define factors that are major sources of variance in the data (S1 Fig). Specifically, we performed Pearson correlation between the PCs and continuous variables (age of samples, vitamin D and testosterone levels) and ANOVA for categorical variables (statin tolerance status, batch, and diabetes status). From this analysis, we identified vitamin D level as a major source of variation and significantly associated with PC1 and PC3. Another source of variation is batch (i.e., the array slide on which a sample was loaded), which was significantly associated with PC2 and PC3. Statin tolerance status was significantly associated with PC3. Based on this, we performed linear regression analysis to evaluate the association between statin myalgia and gene expression following adjustment for vitamin D levels and batch. Given the large number of genes analyzed no single gene association passed stringent genome-wide multiple test correction threshold and we instead applied a nominal cut-off *p* value of < 0.01 to generate a list of genes sufficient to perform pathway analysis to examine if any specific biological function/pathway is overrepresented among the set of small-effect differentially expressed genes (DEGs). The nominal p-value of 0.01 yielded a total of 455 genes. In order to analyze the canonical pathways that may be induced in cases patients, we conducted an Ingenuity IPA and DAVID analysis using this set of DEGs. Significance of differences between groups in demographic and clinical variables was assessed by Student T-Test and Chi-Square analysis with Pearson R for continuous and discrete variables respectively using SAS-JMP Pro (Version 13.1 (SAS Institute, Cary N.C.).

### Gene pathway analysis

For pathway analysis, selected transcripts were subjected to Ingenuity Pathway Analysis (IPA, Qiagen Inc. Redwood City CA). Using the Ingenuity Knowledge Base, a repository of expertly curated biological interactions and functional notations, known functional pathways were tested for enrichment with DEGs. The gene lists were uploaded as a text file and each identifier was mapped to its corresponding gene object. DEGs were assigned to known functional networks based on canonical pathways, relationship to upstream regulators, molecular and cellular functional groupings, and associated network functions. A Fisher’s exact test was used to calculate a *p* value predicting the probability that the assignment of DEGs to that network is explained by chance alone. The canonical network models of DEGs were developed using the top molecules from the molecular and cellular function category in the pathway analysis by IPA (www.ingenuity.com) as outlined in detail previously [13]. The canonical IPA network graphically denotes nodes and edges, or lines, the latter symbolizing biological relations between nodes. In IPA, an assignment of nodes in gene network uses published observations stored in the Ingenuity Pathways Knowledge Base.

To further explore associations of DEG's with functional pathways we evaluated the DEG's using the Functional Annotation Tool of the Database for Annotation, Visualization and Integrated Discovery (DAVID) (Version 6.7, NIAID-NIH, https://david.ncifcrf.gov) [14, 15]. Functional annotation groupings of gene clusters exhibiting significant enrichment among cases (Benjamini-Hochberg adjusted P-value < 0.05) were selected.

To gain additional insight into gene pathways altered by statin exposure, selected individual genes representative of each pathway were examined for their potential role in statin-induced myotoxicity by interrogating a number of databases that include NCBI Gene (www.ncbi.nlm.nih.gov/gene), NCBI Protein (www.ncbi.nlm.nih.gov/protein), Online Mendelian Inheritance in Man (omim.org), PubMed (www.ncbi.nlm.nih.gov/pubmed) and the DAVID Bioinformatics Resources database (Version 6.7, NIAID/NIH; http://david.ncifcrf.gov).

### Genotyping analysis

In order to gain insight into potential genetic modulators of statin myalgia in this small but highly defined population of patients we performed genotyping assays in a subset of the patients with gene expression data (12 cases and 7 controls) using the Affymetrix Axiom Biobank Genotyping Arrays (Affymetrix). This array has over 600,000 markers. For candidate SNP analysis, we determined the frequency of 12 SNPs representing 9 genes that have been previously reported to be associated with statin-related myositis, rhabdomyolysis, and myalgia [16–18]. The allele frequencies in this limited number of highly selected SNPs were compared between cases and controls by Chi-square analysis, and were further compared to expected frequency based upon published genetic databases.

## Results and discussion

### Clinical characteristics of study participants

The demographic and laboratory profiles of patients studied here showed that although the prevalence of hypertension, coronary artery disease and musculoskeletal disease was nominally higher in cases, these did not reach statistical significance due to the relatively small number of patients studied. The prevalence of psychiatric disease and T2DM was comparable between the two groups (Table 1). 25% of cases reported the occurrence of statin myalgia in first-degree relatives (Table 1). The use of vitamin D supplements and testosterone was more frequent among cases, consistent with the clinical practice of adding these medications prior to statin re-challenge [19, 20]. Consistent with the hospital statin formulary guidelines, the majority of cases experienced myalgia with simvastatin, followed by rosuvastatin, atorvastatin and pravastatin (Table 1). Cases had previously experienced muscle symptoms with up to four different statins (average 2.5). Participants reported symptoms of muscle pain, weakness and cramping with statin treatment (Table 1). Symptoms involving leg muscles (thighs and calves) were most frequent however back, neck and shoulder muscle symptoms were also reported (Table 1). In the majority of cases (80%) symptoms were of sufficient severity to interfere with normal daily activities. In all cases, symptoms were bilateral and resolved within 1 week of discontinuing the offending statin and had recurred following re-challenge with a lower dose of the same statin or an alternative statin.

The majority of participants in this study were re-challenged with simvastatin, with no difference in type of statin between cases and controls (Table 1). The average statin intensity [21] also did not differ between cases and controls. 75% of cases experienced recurrence of muscle symptoms during administration of study statin. 30.7% of cases discontinued statin treatment prior to completion of the muscle biopsy (average 9.3 days, range 3–14) due to development of intolerable symptoms.

Consistent with replacement therapy as part of treatment of statin myalgia, mean plasma levels of vitamin D were higher and total testosterone trended higher among cases (Table 1). High sensitivity CRP levels were higher in cases consistent with underlying low-level inflammatory activity (Table 1). Although none of the cases or controls was diagnosed with fibromyalgia, systemic lupus erythematosis, rheumatoid arthritis or other autoimmune disease, low-level elevations in antinuclear antigens (ANA) were detected in two cases and two controls (Table 1). In cases secondary testing revealed the presence of antibody to U1 ribonucleoprotein (RNP) a nuclear antigen released during apoptosis [22] that is associated with mixed connective tissue disease [23]. Plasma Total and Low Density Lipoprotein (LDL) cholesterol were higher in cases reflecting inability to tolerate statin (Table 1).

### Analysis of canonical pathways

Ingenuity Pathway Analysis (IPA) is a system that transforms a list of genes into a set of relevant networks based on records of gene interrelationships maintained in the Ingenuity Pathways Knowledge Base [24, 25] (INGENUITY-QIAGEN Redwood City CA.) IPA shows how genes of interest, in this case DEGs, are biologically related by showing gene-gene interactions thereby identifying significant biologic functions by building networks of interrelated genes [26, 27]. Networks are ranked by P-score, the probability of finding the number of focus genes in each gene network compared to the likelihood of finding the focus genes among a similar number of genes randomly selected from the global gene network (https://www.ingenuity.com/wp-content/themes/ingenuity-qiagen/pdf/ipa/IPA-netgen-algorithm-whitepaper.pdf). A useful feature of IPA is the identification of major canonical pathways with greater than expected enrichment with the genes of interest. IPA analysis identified five canonical pathways enriched with DEGs (Table 2). Enriched pathways included **IGF/PI3-Kinase/Akt Signaling, Cell Cycle, Nerve Growth Factor Signaling, and Cholesterol biosynthesis I and II (dihydrolanosterol) biosynthesis.** Differentially expressed genes in each pathway are shown in Table 2 and in more detail in S2 Table. Genes exhibiting increased expression in Cases patients are listed in ***Bold italics*** and those with decreased expression are listed in *italics*.

**Table 2:** Top 5 canonical pathways identified by IPA *.

Gene Network	P-value	DEGs (Overlap)	Differentially Expressed Genes	Cellular Functions
**Insulin/IGF/PI3K/Akt Signaling**	5.50E-04	9/128 (7.0%)	***ATF2*, *CALM1*, *HRAS*, *PPP3CB*, *RAC1*, *RAF1*, *RELA***, *ITPR2*, *PLEKHA4*	calcium signaling, cell cycle, cell senescence, apoptosis, immune response
**Cell Cycle**	1.16E-03	9/142 (6.3%)	***ARID1A*, *BARD1*, *HDAC3*, *HRAS*, *MRE11A*, *POLR2C*,**, *HDAC7*, *RAD51*, *UBD*	tumor suppression, transcription, cell growth, apoptosis (p53), DNA repair
**Nerve Growth Factor Signaling**	1.33E-03	8/117 (6.8%)	***ATF2*, *HRAS*, *RAF1*, *RAP1A*, *RELA*, *RPS6KB2*, *SMPD4***	GTPase activity, cell cycle, senescence, proliferation, apoptosis
**Cholesterol Biosynthesis I and II (24,25-dihydrolanosterol)**	1.48E-03	3/13 (23.1%)	***FDFT1*, *LSS***, *HSD17B7*	mevalonate pathway, cholesterol biosynthesis, squalene biosynthesis, protein prenylation
				

* ***Bold Italics*** = Upregulated Genes. *Light Italics* = Downregulated genes.

IPA analysis of 455 DEGs (p < 0.01).

These pathways were enriched in DEGs mediating DNA repair (***BARD1*, *MRE11*, *RAD51****)* cell senescence and apoptosis (***HDAC3*, *HDAC7*, *HRAS*, *PPP3CB*, *UBD*, *ATF2*, *RAF1****)* and include several small Ras GTPases (***ATF2*, *HRAS*, *RAP1A****)* (Table 2*)*. The first pathway, the **Insulin/IGF/PI3K/Akt signaling network** regulates diverse cellular functions including metabolism, growth, proliferation cell survival and protein synthesis. Among the up-regulated genes in this network is Calmodulin *(****CALM1****)* a calcium sensor protein that interacts with the ryanodine receptor type 1 calcium channel that mediates release of calcium from the sarcoplasmic reticulum during muscle contraction [28]. In contrast, the other major calcium release receptor inositol 1,4, 5-triphosphate receptor 2 (***ITPR2****)* [29] is down regulated (Table 2). The GTPase HRas proto-oncogene (***HRAS***) that is part of both the PI3K signaling and Cell Cycle Signaling pathways was also upregulated. *HRAS* mediates accumulation of p53 and p16 resulting in cell cycle arrest consistent with cellular senescence [30]. Of note, ***ITPR2*** that triggers calcium release from the endoplasmic reticulum during oncogene-induced senescence allowing mitochondrial calcium accumulation and cell senescence was down regulated [31]. ***PPP3CB*** the catalytic (B) subunit of the protein phosphatase calcineurin (CnA) was also up regulated. Significantly CnA is activated by IGF-1 and participates in recovery from muscle injury [32]. Both the GTPase ***RAC1***, a member of the Rho family of G proteins, and its downstream target ***RAF1*** (*MAP3K*) are up regulated. *RAF1* in turn activates ERK1 and 2 that control gene expression related to cell cycle, apoptosis and cell differentiation (NCBI Gene ID: 5894). ***RELA*** (RELA proto-oncogene, NF-kB subunit) also up regulated is an activating subunit of NF-kB that is involved in TNFα/NF-κB signaling (NCBI Gene ID 5970). NF-kappa B and Rel proteins play a key role in inflammatory response to injury and protection against apoptosis [33]. Up regulated genes in the **Cell Cycle signaling network** include the protein ***BARD1*** that interacts with the tumor suppressor/DNA repair protein BRCA1 (Gene ID 580) that has been proposed to participate in muscle wasting via apoptosis and protein degradation through its ubiquitin ligase activity [34]. Also up regulated is the histone deacetylase ***HDAC3*** that down-regulates p53 to modulate cell growth and apoptosis (Gene ID 8841). In contrast the histone deacetylase ***HDAC7*** is down regulated in muscle of Cases patients (Table 2). Coordinated up-regulation of *HDAC3* and down-regulation of *HDAC7* is associated with skeletal muscle atrophy [35]. Many of the DEGs identified in the PI3K and Cell Cycle Signaling pathways were also identified as part of the **Nerve growth factor pathway** (Table 2). Additional genes identified as up-regulated in this pathway include ***RAP1*** (aka TERF2IP) which encodes *TERF2* interacting protein that is involved in telomere length regulation and apoptosis (NCBI Gene ID 855505), Sphingomyelin phosphodiesterase 4 ***SMPD4*** (aka nSMase3) (NCBI Gene ID 55627) implicated in muscle atrophy as a mediator of TNF-stimulated oxidant activity in skeletal muscle [36], and ribosomal protein S6 kinase B2 (***RPS6KB2***) a serine/threonine kinase that mediates protein synthesis and cell proliferation (NCBI Gene ID 6199). IPA analysis also identified several DEGs in the mevalonate pathway related to **cholesterol and dihydrolanosterol biosynthesis** (Table 2). The up-regulated gene ***FDFT1*** encodes the mevalonate pathway enzyme, farnesyl-diphosphate farnesyltransferase 1 that is responsible for squalene biosynthesis (NCBI Gene ID 2222). Similarly, Lanosterol Synthase (***LS****)* that catalyzes the conversion of (S)-2,3 oxidosqualene to lanosterol is also increased in muscle of cases (NCBI Gene ID 4047). These changes in mevalonate pathway genes likely reflect compensatory up-regulation of distal cholesterol pathway genes in response to inhibition of the proximal cholesterol biosynthesis pathway enzyme HMG-CoA Reductase by statin. Increased expression of these distal pathway genes in muscle of statin myalgia cases suggests a more complete blockade of the mevalonate pathway in muscle either due to increased statin exposure and/or greater statin sensitivity. Similar compensatory upregulation of cholesterol biosynthesis pathway genes has been observed following statin treatment of human skeletal muscle cells in vitro [37]. Blockade of the mevalonate pathway in muscle is significant insofar as the key cholesterol biosynthesis pathway intermediates geranyl pyrophosphate and farnesyl pyrophosphate are needed for prenylation of proteins including the Ras GTPases [38]. Impaired prenylation of these proteins by statins in vitro has been demonstrated to induce apoptosis [39, 40]. Conversely, exogenous geranylgeranol protected cultured myotubes from lovastatin-induced expression of the apoptotic mediator atrogin-1 [41]. Expression of ***HSD17B7*** encoding the isoform of hydroxysteroid dehydrogenase 17B that catalyses the conversion of zymosterone to zymosterol late in the cholesterol biosynthetic pathway was reduced in cases [42].

### Top 5 gene networks identified by IPA

**Fig 1:**
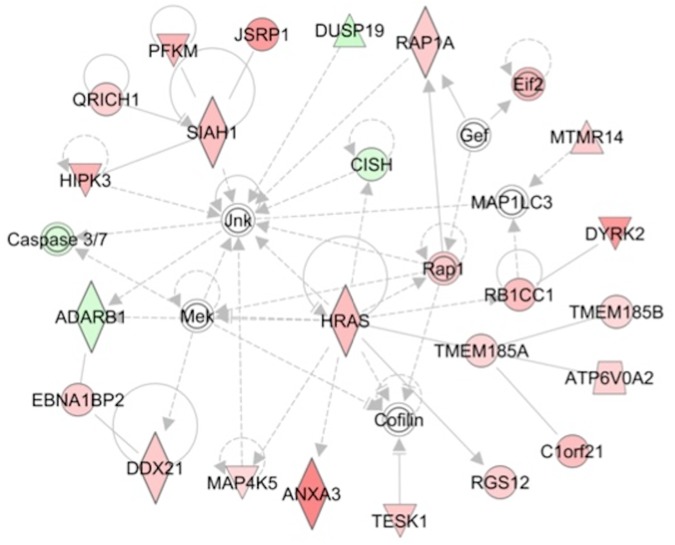
Ingenuity Pathway Analysis (IPA) identifies a network enriched in differentially expressed genes (DEGs). The inter-relationship of DEGs identified as part of a gene network related to organismal injury and skeletal and muscular disorders (Network 4 in Table 3) is depicted visually. Genes exhibiting differential expression in skeletal muscle of cases versus controls are denoted in green (lower expression in cases) and pink/red (higher expression in cases). Regulatory molecules shared by genes within each network some of which are DEGs and some are not are identified as "hubs". Solid lines denote positive interaction and dashed lines inhibitory influences. Genes denoted by circles represent other proteins, triangles represent kinases, inverted triangles represent phosphatases, and diamonds represent enzymes.

In addition to identifying discrete canonical pathways enriched in focus genes, IPA also groups focus genes into multi-pathway gene "networks". The probability that the focus genes are clustered in a given gene network is assessed and ranked by P-score. Table 3 shows the top 5 gene networks formed by DEGs in the muscles of cases. The individual genes and the gene networks themselves are visually presented in Fig 1 and in panels A-D in S2 Fig. In IPA, networks focus genes are organized in a "hub and spoke" orientation. It should be noted however that although a focus gene (i. e., a DEG) may or may not form a "hub" it is connected to multiple focus genes, that provide potential insight into shared regulatory pathways. Up regulated genes are depicted in ***Bold Italics***, down regulated genes in *light Italics* and key non-DEG "Hub" genes associated with DEG's are underlined.

**Table 3:** Five gene networks enriched in differentially expressed genes.

Top Gene Networks Enriched in DEGs	Key "Hub" and "Spoke"Genes*	P -Score
1. Cell Cycle, Apoptosis, Cell Growth	*AKT*, *RPA*, ***BARD1*, *GTF2IRD1*, *XRCC5*, *Mre11*, *MRE11A*, *PPM1A*, *PPM1G*, *TOR1AIP1***, *RAD51*	1E-53
2. Cell Cycle, Nucleic Acid Metabolism, Small Molecule Biochemistry	***NFκB complex*, *AMFR*,** *LRRF1P1*, *PRMT2*	1E-51
3. Gene Expression, Cellular Assembly and Organization	*HISTONE*, *Cbp/p300*, ***E2F4*, *HDAC3*, *thyroid hormone receptor*, *MAPK*, *MAPK6*, *FBXW4*,** *Holo RNA polymerase II*	1E-36
4. Organismal Injury and Abnormalities, Skeletal and Muscular Disorders	*JNK*, *MEK*, *MAP1LC3*, ***HRAS*, *TMEM185A*, *RB1CC1*, *SIAH1*,** *CISH*	1E-34
5. Cellular Assembly and organization, Skeletal and Muscular System Development and Function	*Vegf*, *Mmp*, ***Calpain*, *Calcineurin A*, *CREB*, *YY1*, *ANXA7*, *EPHX4*,** *COL16A1*, *HDAC*	1E-34

* Underlined light italics = regulatory "Node" genes, ***Bold Italics*** = Upregulated Genes. *Light Italics* = Downregulated genes.

Gene network 1 (Cell Cycle, Apoptosis) (Table 3, Panel A in S2 Fig) represents a network centered around Akt involved in cell growth and protein synthesis, and includes the mTORC1 signaling protein DEPTOR and the tumor suppressor protein ***BARD1*** which mediates TP53-induced apoptosis [43]. Another hub gene within network 1 is the protein phosphatase ***PPM1A*** that inhibits the environmental stress-responsive p38 and JNK pathways and activates the tumor suppressor TP53 (NCBI Gene ID 5494). The ***TP53***, identified as an upstream regulator of these networks, is a central controller of cell cycle arrest and apoptosis (Table 4). The down-regulated hub gene ***RAD51*** is involved in homologous recombination and repair of DNA (NCBI Gene ID 5888) as is the linked gene ***XRCC2*** (X-ray repair cross complementing 2) (NCBI Gene ID 7516).

**Table 4:** IPA analysis identifies 5 upstream regulators related to differentially expressed genes (DEGs).

Upstream Regulator	P-value	Functional pathways
**Tumor Repressor Protein 53 (TP53)**	9.90E-05	response to cellular stress, cell cycle arrest, apoptosis, senescence, DNA repair
**Cystatin D (CST5)**	2.74E-04	inhibits lysosomal and secreted cysteine proteases, transcriptional regulation, cytokine secretion
**Monoacylglyerol O-acyltransferase 1 (MOGAT1)**	8.39E-04	diacylglycerol synthesis, triglyceride synthesis
**POU class 2 homeobox 1 (POU2F1)**	9.83E-04	transcriptional regulator, T-cell differentiation
**C-X-C motif chemokine ligand 12 (CXCL12)**	1.49E-03	inflammatory response, leukocyte chemotaxis

The NFκB, a central hub molecule of Gene network 2 (Table 3, Panel B in S2 Fig) is a transcription factor complex that regulates pathways of immunity and inflammation (NCBI Gene ID 4791). This hub is linked to caspase 7 (***CASP7***) a protein that mediates apoptosis (NCBI Gene ID 840) and calpain 3 (***CAPN3***) a protease associated with titin that is involved in limb-girdle muscular dystrophy (NCBI Gene ID 825). Another central hub molecule in network 2 was autocrine motility factor receptor ***AMFR*** (aka GP78) that catalyzes ubiquitination and degradation of proteins (NCBI Gene ID 267). AMFR is indirectly linked to the ryanodine receptor, ***RYR1*** and ***CAMLG*** (NCBI Gene ID 819) that participate in calcium signaling pathways in the muscle. RYR1, a Ca^++^ release channel in the sarcoplasmic reticulum, is associated with central core disease and mini-core myopathy (NCBI Gene ID 6261).

Gene network 3 (Table 3, Panel C in S2 Fig) has as a central hub ***Histone***, a key component of chromatin and gene regulation. Focus genes in this network include transformation/transcription domain associated protein (***TRAPP***) that is a component of histone deacetylase (HDAC) complex (NCBI Gene ID 8295) and ***SFMBT1*** a transcriptional repressor that mediates MyoD-mediated transcriptional silencing [44]. TRAPP in turn is linked to E2F transcription factor 4 (***E2F4***), a suppressor of proliferation-associated genes (NCBI Gene ID 1874) and via E2F4 to histone deacetylase 3 (***HDAC3***) known to down-regulate TP53 related cell growth and apoptosis (NCBI Gene ID 8841).

Gene network 4 (Table 3, Fig 1) is of particular interest with regard to molecular mechanisms of statin myotoxicity. The central hub of this network is ***JNK*** (aka MAPK, mitogen activated protein kinase 8) (NCBI Gene ID 5599) and ***MEK*** (aka mitogen-activated protein kinase kinase 7, MAP2K7) (NCBI Gene ID 5609) that mediate cell responses to pro-inflammatory cytokines and environmental stresses. JNK activation has been linked to statin-induced ER stress and myotoxicity *in vitro* along with enhanced Caspase 3/7 activity [45]. JNK in turn is linked to the E3 ubiquitin ligase SIAH1 that mediates TP53 mediated apoptosis in muscle [46]. A third hub gene is H-Ras proto-oncogene (***HRAS***) that has also been linked to TP53 mediated cell senescence as discussed above (Table 2). The HRAS is linked to Rb1-inducible coiled-coil 1(***RB1CC1***) a potent regulator of the Rb pathway and novel mediator of muscular differentiation [47]; RB1CC1 is also linked to the dual specificity tyrosine phosphorylation regulated kinase 2 (DYRK2) that phosphorylates TP53 to induce apoptosis in response to DNA damage [48].

Calpain and Calcineurin A form the hub genes that represent Gene network 5 (Table 3, Panel D in S2 Fig). The Calpain 3 (***CAPN3***) is a calcium-activated neutral protease that binds to the muscle protein titin (Gene ID 825). Mutations of the Calpain 3 gene are causal for muscular dystrophy, limb-girdle, Type 2A (OMIM #253600). ***Calcineurin*** is a Calcium/calmodulin regulated protein phosphatase that mediates IGF1 stimulated skeletal muscle hypertrophy and a switch to glycolytic metabolism (OMIM #114105) [49, 50]. Calcineurin executes a pro-apoptotic program following exposure of cardiomyocytes to anoxia by promoting translocation of the mitochondrial fission protein Drp1 to mitochondria [51]. Another up regulated node gene is Annexin A7 (***ANXA7***) a membrane binding protein with voltage-sensitive calcium channel activity (Gene ID 310). ANXA7 is linked to the inositol 1,4,5-triphosphate receptor; type 2 *(****ITPR2***) that mediates ER calcium release and mitochondrial calcium accumulation, reactive oxygen species accumulation and cell senescence [31].

### The IPA identifies upstream regulators of differentially expressed genes

Another useful feature of IPA is that it links DEGs to putative upstream regulators as an additional method of identifying discrete signaling pathways that are perturbed under the experimental conditions. As outlined in Table 4, the IPA identified five upstream regulators including **TP53, Cystatin D, MOGAT1, POU2F1**, and C-X-C motif chemokine ligand 12 (**CXCL12**) that could be involved in differential regulation of several key genes and cellular pathways related to statin-intolerance.

In addition to its canonical function as a tumor suppressor, **TP53** is known to mediate the process of muscular atrophy [52]. A second upstream regulator whose target genes are enriched in DEGs is **Cystatin D** (*CST5*) another candidate tumor suppressor gene that is linked to TP53 [53]. **Monoacylglycerol O-acyltransferase 1** is responsible for synthesis of diacylglycerol, the precursor for triacylglycerol and is related to hepatic steatosis and steatohepatitis in obesity [54]. The **POU class 2 homeobox 1** (*POU2F1*; aka *OCT1*) gene encodes a transcription factor that is a known regulator of cellular homeostasis [55]. C-X-C motif chemokine ligand 12 (***CXCL12***) is a chemokine that serves as a potent lymphocyte chemo-attractant [56]. The list of the upstream regulators deduced by IPA is strongly suggestive of pathways of gene expression that underpin cellular stress, and post-inflammatory repair and regeneration.

### Genes exhibiting the greatest degree of over and under expression

To gain further insight into the processes affected by statin re-challenge in Cases, we examined the 20 most highly regulated genes (Tables 5 and 6). The functional roles of these DEGs were deduced by interrogating the NCBI Gene (http://www.ncbi.nlm.nih.gov/gene) and Protein (http://www.ncbi.nlm.nih.gov/protein) databases. Additionally, Online Mendelian Inheritance in Man (OMIM, http://www.ncbi.nlm.nih.gov/omim/) and Pubmed (www.ncbi.nlm.nih.gov/pubmed) databases were searched to identify potential links between DEGs and muscular function. We found that these 20 most highly regulated genes were related to energy metabolism, mitochondrial function, oxidative phosphorylation, inflammatory response and cell proliferation.

**Table 5:** Ingenuity pathway analysis top 10 up-regulated genes.

Gene Symbol	Name/Product	Functional Role**	Fold Change (Int/Tol)*	Nominal P Value = *
***HECTD2-AS1***	HECTD2 antisense RNA 1 (Gene ID 100188947)	Inflammatory response	14.78	0.0961
***UCP3***	Uncoupling Protein 3 (Gene ID 7352)	Muscle energy metabolism, mitochondrial function, protection from oxidative stress	11.61	0.0090
***ALDOA***	Aldolase, fructose-bisphosphate A (Gene ID 226)	Mediates glycolysis, myopathy	10.21	0.0096
***C20orf194***	Chromosome 20 open reading frame 194 (Gene ID 25943)	Unknown	9.91	0.0018
***HOMER3***	Homer scaffolding protein 3 (Gene ID 9454)	Neuronal signaling, T-cell activation, trafficking of amyloid beta peptides.	8.28	0.0002
***DYRK1A***	Dual specificity tyrosine phosphorylation regulated kinase 1A (Gene ID 1859)	Cell proliferation, brain development	8.05	0.0038
***SPATS2L***	Spermatogenesis associated serine rich 2 like (Gene ID 26010)	Response to oxidative stress. Ribosomal biogenesis	7.67	0.0006
***CEP85***	Centrosome protein 85 (Gene ID 64793)	Cell cycle progression (aka CCDC21)	7.31	0.0023
***ZBED1***	Zinc finger BED-type containing 1 (Gene ID 9189)	Transcriptional regulator related to cell proliferation	6.11	0.0003
***ANXA3***	annexing A3 (Gene ID 306)	Signal transduction, cellular growth	5.94	0.0011

* P values calculated by linear regression analysis with adjustment for vitamin D levels.

** Functional role derived from NCBI Protein and Unigene entries.

**Table 6:** Ingenuity pathway analysis top 10 down-regulated genes.

Gene Symbol	Name/Product	Functional Role**	Fold Change (Int/Tol)	Nominal P Value = *
***MFF***	Mitochondrial fission factor (Gene ID 56947)	Mitochondrial and peroxisomal fission,	- 100.00	0.0040
***HLA-DRB6***	Major histocompatibility complex, class II, DR beta 6 (pseudo gene) (Gene ID 3128)	Antigen processing, autoimmunity	-37.04	0.0036
***NNMT***	N-myristoyltransferase 1 (Gene ID 4836)	Protein modification (N-myristoylation), G-protein activation	-13.89	0.0009
***CXCL9***	C-X-C motif chemokine ligand 9 (Gene ID 4283)	T-cell trafficking, lymphocyte chemo attractant	-12.66	< 0.0001
***GBP2***	Guanylate binding protein 2 (Gene ID 2634)	GTPase, immunologic response	-10.99	0.0037
***CFH***	Complement factor H (Gene ID: 3075)	Complement activation, innate immunity	-10.53	0.0096
***TIMP1***	TIMP metallopeptidase inhibitor 1 (Gene ID 7076)	Inhibits matrix metalloproteinases (MMPs), inhibit apoptosis, muscle growth and remodeling	-7.58	0.0097
***LRRFIP1***	LRR binding FLII interacting protein 1 (Gene ID 9208)	Mitogenesis	-6.85	< 0.0001
***GVINP1***	GTPase, very large interferon inducible pseudo gene 1 (Gene ID 387751)	Innate and adaptive cellular immunity	-6.45	0.0050
***NBPF8***	Neuroblastoma breakpoint family member 8 (Gene ID 284565)	Neuronal development	-5.99	0.0028

* P values calculated by linear regression analysis with adjustment for vitamin D levels.

** Functional role derived from NCBI Protein and Unigene entries.

Among the top 10 up regulated genes depicted in Table 5, expression of antisense RNA to the HECT domain E3 ubiquitin protein ligase 2 (***HECTD2-AS1***) was increased 14-fold. HECTD2 is a pro-inflammatory protein that mediates the degradation of the anti-inflammatory protein PIAS1 [57]. Expression of mRNA encoding uncoupling protein 3 (***UCP3***), a mitochondrial anion carrier protein postulated to protect against oxidative stress was expressed in muscle of cases at 12-fold higher levels (Table 5A) [58]. Expression of the glycolytic enzyme aldolase, fructose-bisphosphate A ***ALDOA*** was also increased 10 fold (Table 5A). Genetic deficiency of ALDOA is associated with glycogen storage disease XII, associated with diminished muscle mass and tone, and proximal muscle weakness (OMIM entry #103850). Evidently, ***SPATS2L*** (aka SGNP or Stress Granule and Nucleolar Protein), which is known to protect human myoblasts from oxidative stress [59], was also up regulated (Table 5).

On the other hand, expression of genes related to mitochondrial biogenesis, antigen processing, cellular immunity and adaptive response of muscle were reduced (Table 6). Of these, 4 genes were of potential interest as pertains to statin induced myopathy. Expression of mRNA encoding mitochondrial fission factor ***(MFF)*** was reduced over 100-fold (Table 6). ***MFF*** recruits the cytoplasmic dyanamin-related guanosine triphosphatase Drp1 to the mitochondrial membrane. Knockdown of either ***MFF*** or Drp1 compromises exogenous stimuli-induced mitochondrial fission and apoptosis [60]. Expression of the major histocompatibility complex ***HLA-DRB6*** was also markedly reduced. The ***HLA-DRB*** genotype HLADRB1*1101 is strongly associated with HMG CoA Reductase autoantibody positivity in patients with statin-induced necrotizing autoimmune myopathy [61]. Expression of ***CXCL9*** (aka Mig) a chemo attractant that has been implicated in experimental autoimmune and human myasthenia gravis [62] was also down regulated. Expression of the matrix metalloproteinase inhibitor ***TIMP1*** was reduced in muscle of cases (Table 6). Reduced levels of ***TIMP1*** have been associated with reduced adaptive response to resistance exercise training in older adults [63].

### Functional annotation of DEG's using the Database for Annotation, Visualization, and Integrated Discovery (DAVID)

As a complementary approach to IPA, we also analyzed the set of 455 DEGs using DAVID software (Version 6.7, david.ncifcrf.gov) [14, 15]. A total of 391 of the 455 DEGs were linked to keywords in the Functional Annotation tool (S3 Table). Three functional annotation clusters, containing a greater than expected number of differentially expressed genes, were related to protein catabolism, ubiquitin ligase activation, and nuclear and intracellular organelle lumen (Table 7). The DEGs identified in **cluster 1** contain Caspase 7 (***CASP7***) that mediates apoptosis and programmed cell death, UBX domain protein 1 (***UBXN***) a component of a complex required for proteasome mediated degradation of misfolded proteins, and BRCA1 associated ring domain 1 (***BARD1***) the partner of the DEG *BRAC* identified by IPA analysis (Table 3). The BRAC-BARD1 heterodimer plays a central role in the cell cycle in response to DNA damage (DAVID Bioinformatics Database). **Cluster 2** consists of components of the E3 ubiquitin ligase complex that mediates protein ubiquitination and degradation (***FBXO9*, *HERC2*, *SIAH1*, *ube2g1* and Ube2L6**). **Gene Cluster 3** identified proteins associated with nuclear and intracellular organelle lumen (Table 3). Selected genes from Cluster 3 include those involved in cellular proliferation (***MINA*, *DYRK1A*, *Ppp1cc***) cell cycle regulation (***E2F4***), apoptosis (***DEDD****)*, transcriptional activation (**TAF4, *YY1***) and repression (***IKZF5*, *HDAC3*, *HDAC7***) cholesterol biosynthesis (***PRKAA2***) the catalytic subunit of AMPK and nuclear membrane transport (***NUP54***) (See S3 Table for complete listing).

**Table 7:** Functional annotation clustering of DEGs seen in the muscle of patients experiencing statin myalgia (DAVID functional annotation clustering).

Process	Number of genes	P = *	FDR^#^
***Annotation Cluster 1*: *Protein catabolism (Enrichment Score 6*.*89)***			
Proteolysis involved in protein catabolic process	37	1.8E-5	1.9E-5
Cellular protein catabolic process	37	1.0E-5	2.2E-5
Protein catabolic process	37	1.5E-5	4.8E-5
Modification-dependent protein catabolic process	35	1.6E-5	6.9E-5
Ubi conjugation pathway	31	1.2E-5	1.4E-4
Cellular macromolecule catabolic process	39	5.0E-5	2.6E-4
Proteolysis	42	1.8E-2	1.3E-1
***Annotation Cluster 2*: *Protein Ubiquitination (Enrichment Score 3*.*98)***			
Small conjugating protein ligase activity	14	1.2E-2	3.7E-2
Ubiquitin-protein ligase activity	13	8.4E-3	5.0E-2
Acid-amino acid ligase activity	14	2.9E-2	2.6E-1
***Annotation Cluster 3*: *Nuclear and Organelle Proteins (Enrichment Score 3*.*91)***			
Nuclear lumen	51	4.5E-3	2.0E-2
Organelle lumen	56	2.0E-2	2.7E-1
Intracellular organelle lumen	55	1.6E-2	2.8E-1
Membrane enclosed lumen	56	2.0E-2	4.5E-1

Functional clustering of 391 DEGs using DAVID Bioinformatics Resource (Version 6.7) accessed 9/28/2016 (https://david.ncifcrf.gov).

* P value adjusted for multiple comparisons (Benjamini-Hochberg).

^#^FDR = False Discovery Rate

### Candidate SNP analysis of cases and controls

Susceptibility to statin-induced myopathy has been experimentally linked to specific variants of genes [16, 38, 64, 65] including those related to statin transport and metabolism (*SLCO1B1*, *SLCO2B1*, *ABCC1*, *ABCB1*) [66–70], muscle energy metabolism (*GATM)* [71], pain perception (*HTR3B*, *HTR7*) [72], Ubiquinone/CoQ10 pathway (*CoQ2*) [73] and calcium signaling (*RYR2*) [74]. To determine the prevalence of these known gene variants in our highly characterized population of patients with statin-associated myalgia, we performed SNP analysis using Axiom genotyping array. The minor allele frequency (MAF) for selected polymorphisms with known association with statin myalgia was determined in cases and compared to that of statin-tolerant controls and with known population frequencies (Table 8). Our data corroborated previously reported allele frequency of three genes associated with statin myopathy; these include 2 genes for statin transporters (***SLCO1B1*, *SLCO2B1***) and the gene encoding the ryanadine receptor ***RYR2*** (Table 7). The rs4149056 single nucleotide polymorphism of *SLCO1B1* has been associated with increased risk of statin myalgia [69]. Consistent with this, the rs4149056 variant of the ***SLC01B1*** gene encoding the hepatic uptake transporter OAT1B1 was observed more frequently in patients experiencing statin-associated myalgia (Table 7). The muscle uptake transporter OAT2B1 encoded by the ***SLCO2B1*** gene has been identified as mediating statin uptake and promoting statin toxicity in human skeletal muscle myoblast cells *in vitro [68]*. The rs12422149 polymorphism of ***SLCO2B1*** that has been associated with increased clearance of simvastatin [67] was observed with increased frequency in cases (Table 7). The rs2819742 variant of the Ryanodine Receptor (***RYR***) was also observed with increased frequency in patients with statin-associated myalgia (Table 8). This finding is unexpected insofar as this variant has been associated with reduced risk of rhabdomyolyis [65]. The T allele of C3435T polymorphism (rs1128503) of the efflux transporter ***ABCB1***, that is responsible for intestinal reabsorption of statin as well as efflux of statin from muscle [68], is associated with increased incidence of statin-associated myalgia [75]. In our limited sample of patients, however, we were unable to detect a significant increase in frequency of this polymorphism (Table 8). We were also unable to detect altered frequency of variants of the ***GATM*** gene (Table 8) however the gene variants (rs9806699, rs1719247, rs1346268) associated with protection against myopathy by Mangravite et al [71] were not present on the gene array used in this study. Furthermore the role of ***GATM*** gene variants in statin induced myopathy is at present controversial [76–78]. The significance of these findings requires additional study.

**Table 8:** Observed versus expected minor allele frequency for gene polymorphisms associated with statin myalgia in cases versus controls compared to population frequency.

Gene	SNP ID (Major/minor allele)	Cases MAF^§^ (N = 12)	Controls MAF (N = 7)	Expected MAF ^∞^ HAP MAP (CEU) (N = 90)	Nominal P*
***SLCO1B1***	rs4149056 (C/**T**)	**0.33**	0.00	0.15	**0.039**
	rs2306283 (C/**T)**	0.83	0.43	0.39	0.170
***SLCO2B1***	rs12422149 (G/**A**)	**0.51**	0.08	0.09	**0.01**
*ABCC1*	rs4148330 (A/**G**)	0.92	0.71	0.33	0.313
*ABCB1*	rs1128503 (C/**T**)	0.67	0.43	0.45	0.268
*CoQ2*	rs4693075 (C/**G**)	0.67	0.71	0.34	0.832
***RYR2***	rs2819742 (A/**G**)	**1.00**	0.57	0.40	**0.016**
*HTR3B*	rs2276307 (A/**G**)	0.42	0.43	0.23	0.960
*HTR7*	rs1935349 (A/**G**)	0.17	0.29	0.12	0.832
	rs1176744 (G/**T**)	0.50	0.57	0.25	0.908
*GATM*	rs2453533 (A/**C**)	0.33	0.71	0.29	0.131
	rs1145086 (A/**C/**G/**T)**	0.33	0.71	0.33	0.131

Data are risk allele frequency (%) for SNPs previously associated with statin myalgia and myositis in case control and population studies. MAF is shown for cases and controls along with expected frequency in a population representative of the cohort. Risk Allele is shown in Bold. Differences in allele frequency between cases vs controls were assessed using Chi Square analysis. Nominal P values (Pearson) are provided.

^**§**^ MAF = minor allele frequency.

^**∞**^ Expected minor allele frequency in a European population (HAP-MAP CEU)

* difference in minor allele frequency between cases and controls was assessed by Student T-Test (unadjusted). Nominal p values are presented.

To determine the impact, if any, of these gene variants on synthesis of these proteins we interrogated the RNA micro-array data to determine if any of these genes were differentially expressed. Of the genes examined, none exhibited significant (P < 0.01) over or under expression. This is not unexpected as the majority of SNPs affect function of gene products rather than their synthesis and expression.

### Does gene expression in peripheral blood mononuclear cells provide information regarding the pathophysiology of statin induced myalgia?

Although inflammatory and autoimmune mechanisms have been identified as related to severe forms of statin induced myopathy [79] the extent to which these mechanisms play a role in less severe forms of statin associated myopathy is unclear. To search for evidence of generalized systemic immune activation in response to statin re-challenge we compared gene expression profiles of PBMC's isolated from blood of cases and controls. Although this analysis led to detection of 688 differentially expressed genes (P < 0.01), a hierarchical clustering these DEGs failed to distinguish the cases and controls as unique (S3 Fig). Additionally, it should be noted that the observed changes in gene expression were relatively small (less than 20%) and therefore did not suggest a generalized activation of inflammatory or immune mechanisms in either cohort. Based on these rather limited data we surmise that statin treatment in cases was not associated with a generalized activation of the immune system. Of course, this question needs to be addressed by more rigorous experimentation.

## Conclusions

Several putative mechanisms have been proposed to explain statin-induced myopathy. These include Altered Ca^++^ transport [80], muscle energetics and oxidative stress [81], autoimmunity/inflammation [82], altered statin uptake or metabolism [70, 83] and impaired isoprenylation of proteins [84–86]. Since most of these studies have been focused on statin-induced myositis and rhabdomyolysis, the mechanisms underlying milder forms of statin-induced myopathy are poorly studied. As a result of the lack of objective findings of statin-induced myalgia, many physicians tend to dismiss patient complaints of muscle pain without associated elevation of CPK [87]. To fill this gap, we studied patients with statin-induced myalgia who were undergoing statin re-challenge. Based on these data, we tentatively conclude that statin-induced myalgia was associated with distinct molecular signatures of mitochondrial stress, cell senescence and apoptosis; additionally, muscles of cases elicited gene expression consistent with localized activation of immunity and inflammation, and altered cellular signaling mediated by protein prenylation and RAS-GTPase activation. Thus, we posit that patients with statin myalgia are genetically distinct with respect to their ability to metabolize statins and/or elicit increased end-organ susceptibility.

Insofar as we assessed gene expression in patients with statin-induced myalgia during statin re-challenge, our analyses provide unique and specific information about the changes in skeletal muscle that accompany myalgia symptoms. Previous skeletal muscle biopsy studies in patients with myalgia or overt myositis primarily focused on muscle histopathology with findings of mitochondrial dysfunction, lipid accumulation and fiber atrophy [81, 88], or in cases of statin-associated autoimmune myopathy, overt myocyte necrosis [79]. Two previous muscle biopsy studies examined the effect of statin treatment on muscle gene expression in healthy volunteers treated with high dose statin and with physiologic stress. Laaksonen et. Al. performed gene expression analysis in muscle biopsy specimens from 12 healthy asymptomatic men treated with high dose simvastatin (80 mg/day) and reported activation of pro-apoptotic pathways and genes related to calcium influx [89]. Similarly, Urso et. Al. studied altered gene expression following eccentric exercise in 4 healthy males treated with atorvastatin 80 mg/day and found altered expression of genes related to the ubiquitin proteasome, apoptosis and ion transport pathways [90]. We now extend these findings in patients with statin-associated myalgia. Experimental studies have associated increased expression of the pro-apoptotic ubiquitin proteasome E-3 ligases *MuRF1* (aka *TRIM 63*) and *MAFbx* (aka *Atrogin-1* or *Fbxo32*) with statin myalgia and muscle atrophy [91, 92]. Although we did not detect increased expression of these specific E-3 ligases in the present study, genes of the ubiquitin ligase pathway figured prominently in our analyses, as did genes related to TP53 mediated apoptosis (*BARD1*, *CASP7*, *DEDD*).

A recent study links inhibition of mitochondrial complex III activity by statin with statin-associated myalgia and rhabdomyolysis [93]. Although mitochondrial genes did not figure prominently among our DEGs, individual mitochondrial genes, including mitochondrial fission factor (*MFF)* and uncoupling protein-3 (*UCP3*) exhibited markedly altered expression in muscle of patients with statin-associated myalgia. Furthermore, although genes related to mitochondrial electron transport including succinate dehydrogenase, ubiquinol-cytochrome c reductase, and NADH: ubiquinone oxidoreductase were notably absent from the top differentially expressed genes selected for analysis our findings do not completely exclude a role of altered mitochondrial energy metabolism in the pathogenesis of myalgia associated with statin use insofar as altered substrate levels or enzyme activity would not be detected by our analyses.

Our findings of increased expression of mevalonate pathway enzymes downstream of HMG-CoA Reductase in patients with statin-associated myalgia likely reflects increased exposure of muscle to statin. Under normal conditions systemic exposure to active statin is limited by intestinal re-uptake of statins and by extensive "first pass" hepatic uptake via the portal circulation mediated respectively by the p-glycoprotein MRD1 (encoded by the *ABCB1* gene) [94] and the hepatic transporter OATP1B1 (encoded by the *SLCO1B1* gene) [95]. Single nucleotide polymorphisms of the *ABCB1* and *SLCO1B1* genes have been associated with decreased and increased incidence of myalgia and myositis respectively [70]. Similarly, the uptake transporter SLCO2B1 has been implicated in mediating statin uptake and toxicity in skeletal muscle [68]. Concordant with these observations, gene variants of *SLCO1B1* and *SLCO2B1* were observed with increased frequency in patients with statin-associated myalgia. A gene variant of the ryanodine receptor (*RYR*) that has been associated with statin myotoxicity [65] was also observed with increased frequency.

**Fig 2:**
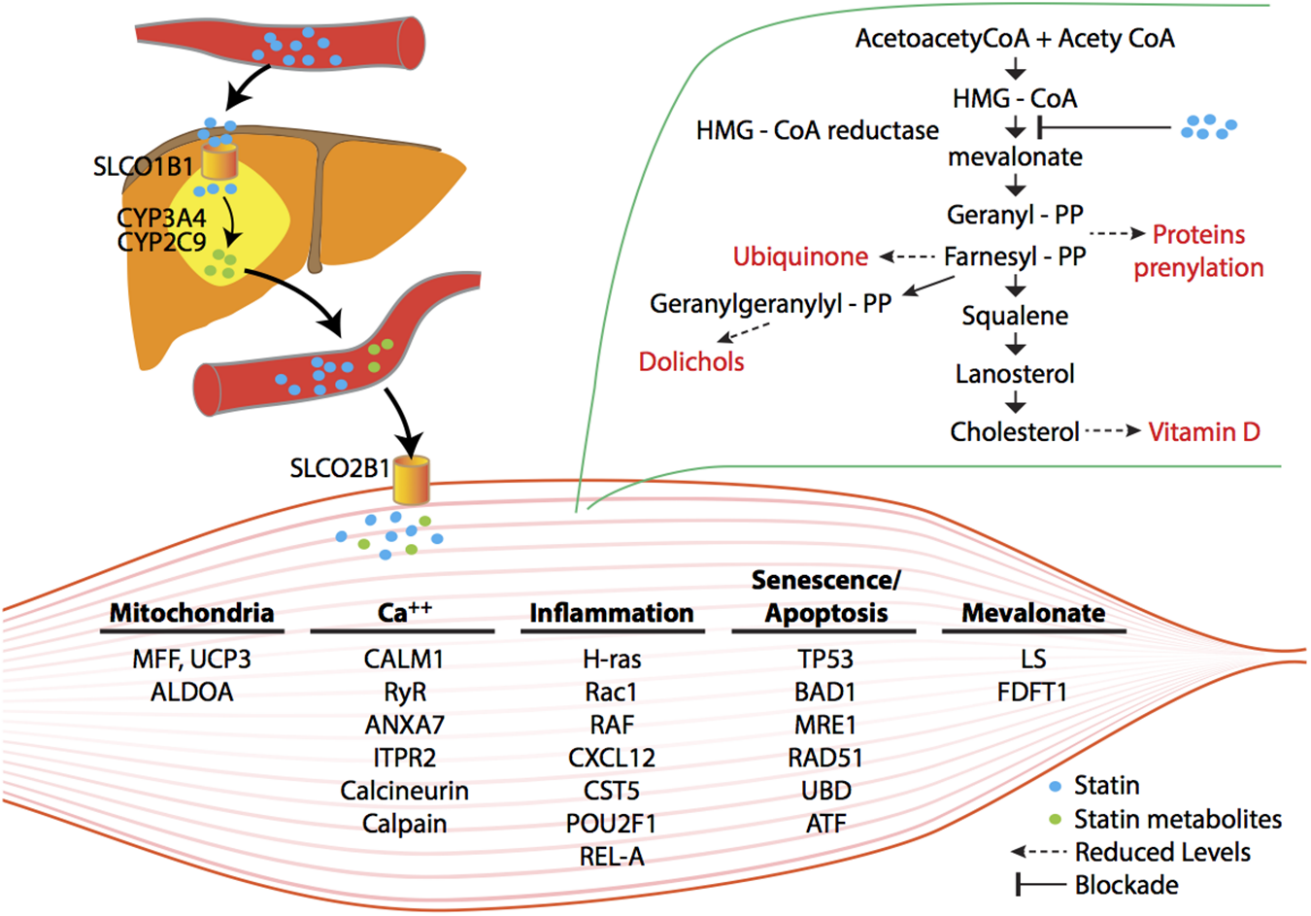
A hypothetical scheme of statin-induced blockade of mevalonate biosynthesis and its consequences for skeletal muscle gene expression and myopathy. Potential differences in inter-organ uptake, metabolism and flux of statins in genetically susceptible patients lead to greater skeletal muscle toxicity. Inhibition of mevalonate and its downstream reaction products result in reduced availability of geranyl-pyrophosphate and farnesyl-pyrophosphate, needed for prenylation/lipidation of signaling proteins. Altered signal transduction pathways re-program skeletal muscle gene expression. Genetic polymorphisms and significantly altered genes that putatively underpin skeletal muscle pathology are indicated. (Modified with permission from Norata GD, Tibolla G, and Catapano AL. Pharmacological Research 88 (2014) 107–113.)

Based on these analyses, we propose a "two-hit" mechanism of statin-induced myalgia. We hypothesize that in some individuals, altered statin uptake or metabolism results in increased exposure of muscle to statin. This, in turn, results in altered mitochondrial function, calcium signaling and activation of pathways of cell senescence and apoptosis (Fig 2). This is likely due at least in part to inhibition of the mevalonate pathway by statin leading to reduced synthesis of key intermediates of the cholesterol biosynthetic pathway needed for prenylation of signaling molecules and altered ubiquitination of protein leading to protein catabolism. This conclusion must be moderated by a number of caveats. First, the cohort of patients profiled here is small and no single gene association passed stringent genome-wide multiple test correction thresholds. To partially circumvent this limitation, we analyzed our gene expression data with IPA and DAVID to identify enrichment of specific gene pathways with differentially expressed genes. Individual genes are presented here only as representative of these functional gene groupings. Second, reflecting current clinical practice, supplementation of vitamin D occurred more frequently among patients with statin myalgia recruited for our study; accordingly, our analyses were adjusted for vitamin D levels. Third, although the candidate gene analysis was limited to a small but well-defined cohort, analysis of a small number of carefully selected SNPs that were previously associated with statin myalgia and myositis coupled with the use of population controls, interpreted in the context of the literature, provides a useful perspective. Fourth, although at the time of publication of this manuscript none of the control participants had developed statin myalgia, statin intolerance can occur even after prolonged statin tolerance. Fifth, these analyses are based on gene expression (mRNA levels) but do not exclude changes in miRNA, protein expression or activity or epigenetic changes. A final caveat is that the gene networks generated by the pathway analyses used here represent statistical associations, not causality. Therefore, findings of pathway analysis, as reported here, must be validated and extended experimentally. These caveats notwithstanding, these analyses provide unique and useful insight into the pathophysiologic and genetic mechanisms underlying this common form of statin induced myopathy and identifies pathways deserving of further study and validation.

## Supporting information

S1 Table Complete listing of genes examined by Affymetrix array. Skeletal muscle gene expression results from over 21,000 probe sets included in the Affymetrix array. Linear regression analysis was performed to test the association between gene expression and statin tolerance following adjustment for vitamin D treatment status. Table includes gene identification, probe ID, gene expression ratio (Case/Control) and nominal P value for difference between statin intolerant and tolerant patients.
(XLSX)

S2 Table Differentially expressed genes (DEGs) in IPA top 5 canonical pathways. Identity, expression ratio (Case/Control) and cellular functions of differentially expressed genes in the top 5 canonical pathways identified by IPA analysis of 455 DEGS (Table 3).
(DOCX)

S3 Table DAVID (Database for Annotation, Visualization, and Integrated Discovery) analysis results for DEGs. Full output of DAVID analysis using DAVID bioinformatics resource version 6.7 ranking functional cluster enrichment with DEGs by P-value. The top cellular processes identified as having greater than expected enrichment in DEGs included pathways related to proteolysis-protein catabolism and the ubiquitin conjugation pathway.
(PDF)

S1 Fig Principal component analysis (PCA) was performed for gene expression data derived from statin-tolerant and statin-intolerant study participants. Initial PCA analysis of 23 samples identified 3 outliers that were removed from fruther analysis. PC analysis of the remaining 20 samples (shown here) showed that PC1 explained the highest amount of variance. In bivariate analysis Vitamin D level was moderately correlated with PC1 and PC3. Batch (i.e. the array slide on which a sample was loaded was signficantly associated with PC2 and PC3. Statin tolerance status was significantly associated with PC3. Based upon the results of this analysis linear regression analysis was performed to evaluate the association between statin intolerance and gene expression with adjustment for vitamin D levels and batch effect.
(TIF)

S2 Fig IPA analysis organizes differentially expressed genes into five major gene networks based upon ingenuity pathway database search. Genes exhibiting differential expression in skeletal muscle of statin intolerant vs tolerant study participants are denoted in green (lower expression in statin intolerant) and pink/red (higher expression in statin intolerant study subjects). Regulatory molecules shared by genes within each network some of which are DEGs and some are not are identified as "hubs". Solid lines denote positive interaction and dashed lines inhibitory influences. Genes denoted by circles represent other proteins, triangles represent kinases, inverted triangles represent phosphatases, and diamonds represent enzymes. Gene networks identified are (Panel A): Network 1, Cell Cycle/Cancer (Panel B): Network 2, Cell Cycle/Small Molecule Biochemistry (Panel C): Network 3, Gene Expression/Cellular Assembly and Organization (Panel D): Network 5, Skeletal and Muscular Development and Function.
(TIF)

S3 Fig Hierachical analysis of 35,812 genes in peripheral blood mononuclear cells of statin intolerant (IN) and statin tolerant (T) study participants. Hierarchical analysis shows no clear differentiation in gene expression in PBMCs isolated from statin tolerant and intolerant subjects.
(TIF)
