# Supplementary material for: Patients experiencing statin-induced myalgia exhibit a unique program of skeletal muscle gene expression following statin re-challenge
Source: PLoS One. 2017 Aug 3;12(8):e0181308. doi: 10.1371/journal.pone.0181308 (PMC5542661; doi:10.1371/journal.pone.0181308)
Supplement: S2 Table — Identity, expression ratio (Case/Control) and cellular functions of differentially expressed genes in the top 5 canonical pathways identified by IPA analysis of 455 DEGS (Table 3). (DOCX) [file pone.0181308.s002.docx]

S2 Table: Differentially expressed genes (DEGs) in IPA top Canonical Pathways.

| Gene Network | Gene Symbol | Gene Name | Expression   (Int/Tol) | Cellular Function(s) |
| --- | --- | --- | --- | --- |
| insulin/IGF/PI3K/Akt signaling | **ATF2** | **activating transcription factor 2** | **3.318** | **GTPase activity, cell cycle, senescence** |
|  | **CALM1** | **Calmodulin 1 (phosphorylase)** | **3.003** | **calcium sensor, ryanodine receptor function, muscle contraction** |
|  | **HRAS** | **Harvey rat sarcoma viral** | **3.178** | **GTPase activity, cell cycle, senescence** |
|  | ITPR2 | inositol 1,4,5-triphosphate receptor type 2 | -2.591 | IP3 mediated intracellular calcium release,,target for anti-apoptotic proteins. |
|  | PLEKHA4 | pleckstrin homology domain A4 | -3.953 | phosphatidylinositol 3-phosphate binding |
|  | **PPP3CB** | **protein phosphatase 3 catalytic**  **(*CALB*: Calcineurin)** | **2.247** | **myoblast recruitment, recovery from muscle injury.** |
|  | **RAC1** | **ras-related C3 botulinum toxin** | **2.187** | **RAS GTPase, cell growth, cytoskeleton** |
|  | **RAF1** | **Raf-1 proto-oncogene** | **3.317** | **MAP3K, ERK activation, apoptosis** |
|  | **RELA** | **RELA proto-oncogene, NF-kβ** | **1.726** | **immune response, cell differentiation, apoptosis** |
| Cell Cycle, Senescence, Apoptosis | **ARID1Ah** | **AT-rich interaction domain 1A** | **2.720** | **transcriptional activation, chromatin remodeling, cardiac muscle differentiation** |
|  | **BARD1** | **BRCA1 associated RING Domain** | **4.839** | **Cellular response to DNA damage stimulus, positive regulation of apoptosis, protein catabolism** |
|  | HDAC3 | Histone deacetylase 3 | 1.609 | p53 regulation, cell growth and apoptosis |
|  | **HDAC7** | **histone deacetylase 7** | **-2.941** | **skeletal muscle atrophy** |
|  | **HRAS** | **Harvey rat sarcoma viral** | **3.178** | **GTPase activity, cell cycle, senescence** |
|  | **MRE11A** | **MRE11 homolog A, double strand** | **1.996** | **DNA repair, homologous recombination, telemere length maintenance** |
|  | **POLR2C** | **polymerase (RNA) II subunit C** | **4.572** | **subunit of RNA polymerase II, mRNA synthesis** |
|  | RAD51 | RAD51 recombinase | -2.717 | homologous recombination and repair of DNA, BRCA1 binding, cellular response to DNA damage |
|  | UBD | ubiquitin D | -2.398 | apoptosis, proteolysis, protein ubiquitination, |
| Nerve Growth Factor Signaling | **ATF2** | **activating transcription factor 2** | **3.318** | **GTPase activity, cell cycle, senescence** |
|  | **HRAS** | **Harvey rat sarcoma vital** | **3.178** | **GTPase activity, cell cycle, senescence** |
|  | **RAC1** | **ras-related C3 botulinum toxin** | **2.187** | **Ras GTPase, cell growth, cytoskeleton** |
|  | **RAF1** | **Raf-1 proto-oncogene** | **3.317** | **MAP3K, ERK activation, apoptosis** |
|  | **RAP1A** | **RAP1A member of RAS oncogene family** | **2.515** | **Ras GTPase, cell proliferation,** |
|  | **RELA** | **RELA proto-oncogene, NF-kβ** | **1.726** | **Chromatin remodeling, transcriptional activation** |
|  | **RPS6KB2** | **ribosomal protein S6 kinase B2** | **2.520** | **protein synthesis and cell proliferation** |
|  | **SMPD4** | **sphingomyelin phosphodiesterase 4** | **1.761** | **sphingolipid signaling, oxidative stress, skeletal muscle atrophy** |
| Cholesterol Biosynthesis I and II | **FDFT1** | **farnesyl-diphosphate** | **2.526** | **mevalonate pathway, Squalene biosynthesis, protein prenylation** |
|  | HSD17B7 | hydroxysteroid 17-β | -2.950 | cholesterol biosynthesis, sex steroid biosynthesis |
|  | **LSS** | **lanosterol synthase 2,3** | **2.214** | **Cholesterol biosynthesis pathway** |
|  |  |  |  |  |

***Bold font** = Genes upregulated in statin intolerant patients. Light font = Downregulated genes.

IPA analysis of 455 DEGs (p < 0.01).
