# Supplementary material for: Patients experiencing statin-induced myalgia exhibit a unique program of skeletal muscle gene expression following statin re-challenge
Source: PLoS One. 2017 Aug 3;12(8):e0181308. doi: 10.1371/journal.pone.0181308 (PMC5542661; doi:10.1371/journal.pone.0181308)
Supplement: S3 Table — Full output of DAVID analysis using DAVID bioinformatics resource version 6.7 ranking functional cluster enrichment with DEGs by P-value. The top cellular processes identified as having greater than expected enrichment in DEGs included pathways related to proteolysis-protein catabolism and the ubiquitin conjugation pathway. (PDF) [file pone.0181308.s003.pdf]

DAVID Bioinformatics Resources 6.7
National Institute of Allergy and Infectious Diseases (NIAID), NIH

*** Announcing DAVID 6.8 Beta with updated Knowledgebase (more info). You may explore the new version at david-d.ncifcrf.gov. *** DAVID 6.8 will become the production version on October 17, 2016 at this URL. DAVID 6.7 will be available on our development site until at least January 15, 2017. ***

Functional Annotation Clustering

Help and Manual

Current Gene List: List_1
Current Background: Homo sapiens
391 DAVID IDs

Options **Classification Stringency** High

Rerun using options Create Sublist

118 Cluster(s)

Download File

Category	Term		Count	P_Value	Benjamini	FDR
Annotation Cluster 1	Enrichment Score: 6.89	G	Count	P_Value	Benjamini	FDR
GOTERM_BP_FAT	proteolysis involved in cellular protein catabolic process	RT	37	1.1E-8	1.8E-5	1.9E-5
GOTERM_BP_FAT	cellular protein catabolic process	RT	37	1.3E-8	1.0E-5	2.2E-5
GOTERM_BP_FAT	protein catabolic process	RT	37	2.9E-8	1.5E-5	4.8E-5
GOTERM_BP_FAT	modification-dependent protein catabolic process	RT	35	4.1E-8	1.6E-5	6.9E-5
GOTERM_BP_FAT	modification-dependent macromolecule catabolic process	RT	35	4.1E-8	1.6E-5	6.9E-5
SP_PIR_KEYWORDS	ubl conjugation pathway	RT	31	1.0E-7	1.2E-5	1.4E-4
GOTERM_BP_FAT	cellular macromolecule catabolic process	RT	39	1.6E-7	5.0E-5	2.6E-4
GOTERM_BP_FAT	macromolecule catabolic process	RT	39	9.8E-7	2.6E-4	1.6E-3
GOTERM_BP_FAT	proteolysis	RT	42	8.0E-5	1.8E-2	1.3E-1
Annotation Cluster 2	Enrichment Score: 3.98	G	Count	P_Value	Benjamini	FDR
GOTERM_MF_FAT	small conjugating protein ligase activity	RT	14	2.6E-5	1.2E-2	3.7E-2
GOTERM_MF_FAT	ubiquitin-protein ligase activity	RT	13	3.5E-5	8.4E-3	5.0E-2
GOTERM_MF_FAT	acid-amino acid ligase activity	RT	14	1.8E-4	2.9E-2	2.6E-1
GOTERM_MF_FAT	ligase activity, forming carbon-nitrogen bonds	RT	14	7.0E-4	6.5E-2	1.0E0
Annotation Cluster 3	Enrichment Score: 3.91	G	Count	P_Value	Benjamini	FDR
GOTERM_CC_FAT	nuclear lumen	RT	51	1.5E-5	4.5E-3	2.0E-2
GOTERM_CC_FAT	organelle lumen	RT	56	2.0E-4	2.0E-2	2.7E-1
GOTERM_CC_FAT	intracellular organelle lumen	RT	55	2.1E-4	1.6E-2	2.8E-1
GOTERM_CC_FAT	membrane-enclosed lumen	RT	56	3.4E-4	2.0E-2	4.5E-1
Annotation Cluster 4	Enrichment Score: 2.93	G	Count	P_Value	Benjamini	FDR
GOTERM_MF_FAT	metal ion binding	RT	107	8.9E-4	6.9E-2	1.3E0
GOTERM_MF_FAT	cation binding	RT	107	1.3E-3	7.3E-2	1.8E0
GOTERM_MF_FAT	ion binding	RT	108	1.4E-3	7.4E-2	2.1E0
Annotation Cluster 5	Enrichment Score: 2.77	G	Count	P_Value	Benjamini	FDR
GOTERM_CC_FAT	myofibril	RT	9	1.2E-3	5.7E-2	1.6E0
GOTERM_CC_FAT	contractile fiber part	RT	9	1.3E-3	5.5E-2	1.8E0
GOTERM_CC_FAT	contractile fiber	RT	9	2.1E-3	7.4E-2	2.7E0
GOTERM_CC_FAT	sarcomere	RT	8	2.5E-3	8.0E-2	3.3E0
Annotation Cluster 6	Enrichment Score: 2.74	G	Count	P_Value	Benjamini	FDR
SP_PIR_KEYWORDS	zinc-finger	RT	54	7.1E-4	4.2E-2	9.7E-1
GOTERM_MF_FAT	transition metal ion binding	RT	77	1.3E-3	8.2E-2	1.8E0
SP_PIR_KEYWORDS	zinc	RT	63	2.1E-3	7.2E-2	2.8E0
GOTERM_MF_FAT	zinc ion binding	RT	63	6.0E-3	2.1E-1	8.3E0
Annotation Cluster 7	Enrichment Score: 2.27	G	Count	P_Value	Benjamini	FDR
GOTERM_BP_FAT	protein ubiquitination	RT	9	3.5E-3	5.0E-1	5.7E0
GOTERM_BP_FAT	protein modification by small protein conjugation or removal	RT	10	6.5E-3	6.9E-1	1.0E1
GOTERM_BP_FAT	protein modification by small protein conjugation	RT	9	6.6E-3	6.5E-1	1.0E1
Annotation Cluster 8	Enrichment Score: 2.1	G	Count	P_Value	Benjamini	FDR

Annotation Cluster 1	Enrichment Score: 6.89	G		Count	P_Value	Benjamini	FDR
UP_SEQ_FEATURE	domain:SOCS box	RT		5	6.6E-3	8.7E-1	1.0E1
INTERPRO	SOCS protein, C-terminal	RT		5	7.1E-3	9.9E-1	1.0E1
SMART	SOCS	RT		5	1.1E-2	8.2E-1	1.2E1
Annotation Cluster 9	Enrichment Score: 1.93	G		Count	P_Value	Benjamini	FDR
GOTERM_CC_FAT	mitochondrial outer membrane	RT		7	7.0E-3	1.6E-1	9.0E0
GOTERM_CC_FAT	organelle outer membrane	RT		7	1.4E-2	2.2E-1	1.7E1
GOTERM_CC_FAT	outer membrane	RT		7	1.6E-2	2.1E-1	2.0E1
Annotation Cluster 10	Enrichment Score: 1.8	G		Count	P_Value	Benjamini	FDR
INTERPRO	Cyclin-like F-box	RT		6	1.4E-2	9.0E-1	1.9E1
UP_SEQ_FEATURE	domain:F-box	RT		6	1.4E-2	9.2E-1	2.1E1
SMART	FBOX	RT		6	2.0E-2	6.7E-1	2.2E1
Annotation Cluster 11	Enrichment Score: 1.78	G		Count	P_Value	Benjamini	FDR
SP_PIR_KEYWORDS	ank repeat	RT		12	8.3E-3	2.1E-1	1.1E1
INTERPRO	Ankyrin	RT		12	9.5E-3	9.6E-1	1.3E1
UP_SEQ_FEATURE	repeat:ANK 1	RT		11	1.7E-2	9.1E-1	2.5E1
UP_SEQ_FEATURE	repeat:ANK 2	RT		11	1.8E-2	8.9E-1	2.5E1
SMART	ANK	RT		12	2.0E-2	8.1E-1	2.2E1
UP_SEQ_FEATURE	repeat:ANK 3	RT		9	4.0E-2	9.5E-1	4.8E1
Annotation Cluster 12	Enrichment Score: 1.75	G		Count	P_Value	Benjamini	FDR
GOTERM_CC_FAT	sarcoplasmic reticulum membrane	RT		4	3.7E-3	9.6E-2	4.8E0
GOTERM_CC_FAT	sarcoplasmic reticulum	RT		4	3.7E-2	3.2E-1	3.9E1
GOTERM_CC_FAT	sarcoplasm	RT		4	4.2E-2	3.5E-1	4.3E1
Annotation Cluster 13	Enrichment Score: 1.7	G		Count	P_Value	Benjamini	FDR
GOTERM_BP_FAT	regulation of apoptosis	RT		27	1.8E-2	8.8E-1	2.6E1
GOTERM_BP_FAT	regulation of programmed cell death	RT		27	2.0E-2	8.7E-1	2.9E1
GOTERM_BP_FAT	regulation of cell death	RT		27	2.1E-2	8.6E-1	3.0E1
Annotation Cluster 14	Enrichment Score: 1.68	G		Count	P_Value	Benjamini	FDR
GOTERM_BP_FAT	telomere maintenance	RT		4	2.0E-2	8.9E-1	2.9E1
KEGG_PATHWAY	Non-homologous end-joining	RT		3	2.1E-2	7.0E-1	2.1E1
GOTERM_BP_FAT	telomere organization	RT		4	2.2E-2	8.6E-1	3.1E1
Annotation Cluster 15	Enrichment Score: 1.62	G		Count	P_Value	Benjamini	FDR
UP_SEQ_FEATURE	zinc finger region:RING-type	RT		11	9.4E-3	9.0E-1	1.4E1
INTERPRO	Zinc finger, RING-type, conserved site	RT		13	1.2E-2	9.3E-1	1.7E1
INTERPRO	Zinc finger, RING-type	RT		12	4.0E-2	9.6E-1	4.5E1
SMART	RING	RT		12	7.6E-2	8.4E-1	6.2E1
Annotation Cluster 16	Enrichment Score: 1.61	G		Count	P_Value	Benjamini	FDR
GOTERM_CC_FAT	integrator complex	RT		3	2.4E-2	2.5E-1	2.8E1
GOTERM_BP_FAT	snRNA processing	RT		3	2.5E-2	8.5E-1	3.5E1
GOTERM_BP_FAT	snRNA metabolic process	RT		3	2.5E-2	8.5E-1	3.5E1
Annotation Cluster 17	Enrichment Score: 1.57	G		Count	P_Value	Benjamini	FDR
GOTERM_CC_FAT	endoplasmic reticulum part	RT		14	1.5E-2	2.1E-1	1.8E1
GOTERM_CC_FAT	endoplasmic reticulum membrane	RT		11	3.2E-2	2.9E-1	3.5E1
GOTERM_CC_FAT	nuclear envelope-endoplasmic reticulum network	RT		11	4.3E-2	3.5E-1	4.4E1
Annotation Cluster 18	Enrichment Score: 1.49	G		Count	P_Value	Benjamini	FDR
UP_SEQ_FEATURE	repeat:MORN 4	RT		3	1.9E-2	8.6E-1	2.7E1
UP_SEQ_FEATURE	repeat:MORN 3	RT		3	3.0E-2	9.2E-1	3.9E1
UP_SEQ_FEATURE	repeat:MORN 2	RT		3	3.5E-2	9.3E-1	4.4E1
UP_SEQ_FEATURE	repeat:MORN 1	RT		3	3.5E-2	9.3E-1	4.4E1
INTERPRO	MORN motif	RT		3	3.6E-2	9.7E-1	4.2E1
SMART	MORN	RT		3	4.5E-2	8.5E-1	4.3E1
Annotation Cluster 19	Enrichment Score: 1.42	G		Count	P_Value	Benjamini	FDR
GOTERM_BP_FAT	negative regulation of apoptosis	RT		14	3.6E-2	8.8E-1	4.6E1
GOTERM_BP_FAT	negative regulation of programmed cell death	RT		14	3.9E-2	8.8E-1	4.9E1
GOTERM_BP_FAT	negative regulation of cell death	RT		14	4.0E-2	8.7E-1	5.0E1

	Category	Term			Count	P_Value	Benjamini	FDR
	Annotation Cluster 1	Enrichment Score: 6.89	G		Count	P_Value	Benjamini	FDR
	Annotation Cluster 20	Enrichment Score: 1.4	G		Count	P_Value	Benjamini	FDR
☐	SP_PIR_KEYWORDS	chromatin regulator	RT		11	9.9E-3	2.2E-1	1.3E1
☐	GOTERM_BP_FAT	chromatin modification	RT		11	6.3E-2	9.3E-1	6.6E1
☐	GOTERM_BP_FAT	chromatin organization	RT		13	1.0E-1	9.6E-1	8.3E1
	Annotation Cluster 21	Enrichment Score: 1.34	G		Count	P_Value	Benjamini	FDR
☐	UP_SEQ_FEATURE	nucleotide phosphate-binding region:GTP	RT		13	1.5E-2	9.0E-1	2.2E1
☐	SP_PIR_KEYWORDS	gtp-binding	RT		13	3.1E-2	3.7E-1	3.5E1
☐	GOTERM_MF_FAT	GTP binding	RT		13	6.8E-2	7.4E-1	6.3E1
☐	GOTERM_MF_FAT	guanyl ribonucleotide binding	RT		13	7.8E-2	7.7E-1	6.9E1
☐	GOTERM_MF_FAT	guanyl nucleotide binding	RT		13	7.8E-2	7.7E-1	6.9E1
	Annotation Cluster 22	Enrichment Score: 1.32	G		Count	P_Value	Benjamini	FDR
☐	SP_PIR_KEYWORDS	DNA damage	RT		10	2.1E-2	2.9E-1	2.5E1
☐	SP_PIR_KEYWORDS	dna repair	RT		9	3.6E-2	4.1E-1	4.0E1
☐	GOTERM_BP_FAT	DNA repair	RT		11	7.6E-2	9.5E-1	7.3E1
☐	GOTERM_BP_FAT	response to DNA damage stimulus	RT		13	9.3E-2	9.6E-1	8.0E1
	Annotation Cluster 23	Enrichment Score: 1.25	G		Count	P_Value	Benjamini	FDR
☐	GOTERM_MF_FAT	double-stranded RNA binding	RT		4	2.7E-2	5.8E-1	3.2E1
☐	INTERPRO	Double-stranded RNA binding	RT		3	7.3E-2	9.7E-1	6.8E1
☐	SMART	DSRM	RT		3	8.9E-2	8.2E-1	6.8E1
	Annotation Cluster 24	Enrichment Score: 1.15	G		Count	P_Value	Benjamini	FDR
☐	GOTERM_BP_FAT	regulation of I-kappaB kinase/NF-kappaB cascade	RT		7	2.5E-2	8.6E-1	3.4E1
☐	GOTERM_BP_FAT	positive regulation of I-kappaB kinase/NF-kappaB cascade	RT		6	5.3E-2	9.0E-1	6.0E1
☐	GOTERM_BP_FAT	positive regulation of protein kinase cascade	RT		6	2.7E-1	9.9E-1	1.0E2
	Annotation Cluster 25	Enrichment Score: 1.13	G		Count	P_Value	Benjamini	FDR
☐	GOTERM_CC_FAT	insoluble fraction	RT		24	4.5E-2	3.4E-1	4.6E1
☐	GOTERM_CC_FAT	membrane fraction	RT		23	5.3E-2	3.8E-1	5.2E1
☐	GOTERM_CC_FAT	cell fraction	RT		26	1.7E-1	7.2E-1	9.2E1
	Annotation Cluster 26	Enrichment Score: 1.06	G		Count	P_Value	Benjamini	FDR
☐	INTERPRO	Ubiquitin-associated/translation elongation factor EF1B, N-terminal, eukaryote	RT		4	5.3E-2	9.7E-1	5.6E1
☐	SMART	UBA	RT		4	7.0E-2	8.6E-1	5.9E1
☐	INTERPRO	Ubiquitin-associated/translation elongation factor EF1B, N-terminal	RT		3	1.8E-1	1.0E0	9.5E1
	Annotation Cluster 27	Enrichment Score: 1.01	G		Count	P_Value	Benjamini	FDR
☐	GOTERM_BP_FAT	transcription, DNA-dependent	RT		11	8.7E-2	9.5E-1	7.8E1
☐	GOTERM_BP_FAT	RNA biosynthetic process	RT		11	9.3E-2	9.6E-1	8.1E1
☐	GOTERM_BP_FAT	transcription from RNA polymerase II promoter	RT		9	1.2E-1	9.7E-1	8.8E1
	Annotation Cluster 28	Enrichment Score: 1	G		Count	P_Value	Benjamini	FDR
☐	SP_PIR_KEYWORDS	nucleotide-binding	RT		43	6.8E-2	5.2E-1	6.2E1
☐	GOTERM_MF_FAT	nucleotide binding	RT		54	9.2E-2	8.2E-1	7.5E1
☐	GOTERM_MF_FAT	ribonucleotide binding	RT		45	1.0E-1	7.9E-1	7.8E1
☐	GOTERM_MF_FAT	purine ribonucleotide binding	RT		45	1.0E-1	7.9E-1	7.8E1
☐	GOTERM_MF_FAT	purine nucleotide binding	RT		45	1.6E-1	8.7E-1	9.2E1
	Annotation Cluster 29	Enrichment Score: 0.99	G		Count	P_Value	Benjamini	FDR
☐	GOTERM_BP_FAT	cell death	RT		22	7.6E-2	9.4E-1	7.3E1
☐	GOTERM_BP_FAT	death	RT		22	7.8E-2	9.4E-1	7.4E1
☐	GOTERM_BP_FAT	apoptosis	RT		18	1.3E-1	9.7E-1	9.0E1
☐	GOTERM_BP_FAT	programmed cell death	RT		18	1.4E-1	9.8E-1	9.2E1
	Annotation Cluster 30	Enrichment Score: 0.93	G		Count	P_Value	Benjamini	FDR
☐	GOTERM_CC_FAT	mitochondrial membrane	RT		13	7.0E-2	4.6E-1	6.2E1
☐	GOTERM_CC_FAT	mitochondrial envelope	RT		13	9.9E-2	5.5E-1	7.5E1
☐	GOTERM_CC_FAT	mitochondrial part	RT		15	2.3E-1	7.8E-1	9.7E1
	Annotation Cluster 31	Enrichment Score: 0.92	G		Count	P_Value	Benjamini	FDR
☐	UP_SEQ_FEATURE	repeat:ANK 8	RT		4	5.0E-2	9.7E-1	5.7E1
☐	UP_SEQ_FEATURE	repeat:ANK 7	RT		4	1.1E-1	1.0E0	8.4E1

	Category	Term		Count	P_Value	Benjamini	FDR
	Annotation Cluster 1	Enrichment Score: 6.89	G	Count	P_Value	Benjamini	FDR
☐	UP_SEQ_FEATURE	repeat:ANK 6	RT	5	1.1E-1	1.0E0	8.5E1
☐	UP_SEQ_FEATURE	repeat:ANK 9	RT	3	1.7E-1	1.0E0	9.5E1
☐	UP_SEQ_FEATURE	repeat:ANK 5	RT	5	2.6E-1	1.0E0	9.9E1
	Annotation Cluster 32	Enrichment Score: 0.91	G	Count	P_Value	Benjamini	FDR
☐	GOTERM_BP_FAT	negative regulation of protein kinase activity	RT	5	1.1E-1	9.7E-1	8.6E1
☐	GOTERM_BP_FAT	negative regulation of kinase activity	RT	5	1.2E-1	9.7E-1	8.8E1
☐	GOTERM_BP_FAT	negative regulation of transferase activity	RT	5	1.4E-1	9.8E-1	9.2E1
	Annotation Cluster 33	Enrichment Score: 0.9	G	Count	P_Value	Benjamini	FDR
☐	SP_PIR_KEYWORDS	Transcription	RT	52	5.5E-2	4.7E-1	5.4E1
☐	SP_PIR_KEYWORDS	transcription regulation	RT	49	1.0E-1	6.3E-1	7.7E1
☐	GOTERM_BP_FAT	transcription	RT	52	1.4E-1	9.8E-1	9.2E1
☐	GOTERM_BP_FAT	regulation of transcription	RT	59	3.1E-1	9.9E-1	1.0E2
	Annotation Cluster 34	Enrichment Score: 0.87	G	Count	P_Value	Benjamini	FDR
☐	UP_SEQ_FEATURE	domain:JmjC	RT	3	1.2E-1	1.0E0	8.7E1
☐	INTERPRO	Transcription factor jumonji/aspartyl beta-hydroxylase	RT	3	1.3E-1	9.9E-1	8.8E1
☐	SMART	JmjC	RT	3	1.6E-1	9.4E-1	8.7E1
	Annotation Cluster 35	Enrichment Score: 0.87	G	Count	P_Value	Benjamini	FDR
☐	GOTERM_MF_FAT	purine NTP-dependent helicase activity	RT	6	4.5E-2	6.7E-1	4.8E1
☐	GOTERM_MF_FAT	ATP-dependent helicase activity	RT	6	4.5E-2	6.7E-1	4.8E1
☐	SP_PIR_KEYWORDS	helicase	RT	6	1.3E-1	6.9E-1	8.4E1
☐	INTERPRO	DNA/RNA helicase, DEAD/DEAH box type, N-terminal	RT	4	1.3E-1	9.9E-1	8.7E1
☐	GOTERM_MF_FAT	helicase activity	RT	6	1.4E-1	8.6E-1	8.9E1
☐	UP_SEQ_FEATURE	domain:Helicase C-terminal	RT	5	1.6E-1	1.0E0	9.4E1
☐	INTERPRO	DNA/RNA helicase, C-terminal	RT	5	1.7E-1	1.0E0	9.4E1
☐	INTERPRO	Helicase, superfamily 1 and 2, ATP-binding	RT	5	1.7E-1	1.0E0	9.4E1
☐	UP_SEQ_FEATURE	domain:Helicase ATP-binding	RT	5	1.7E-1	1.0E0	9.6E1
☐	INTERPRO	DEAD-like helicase, N-terminal	RT	5	1.8E-1	1.0E0	9.5E1
☐	SMART	HELICc	RT	5	2.2E-1	9.7E-1	9.5E1
☐	SMART	DEXDc	RT	5	2.4E-1	9.7E-1	9.6E1
	Annotation Cluster 36	Enrichment Score: 0.87	G	Count	P_Value	Benjamini	FDR
☐	INTERPRO	Ubiquitin	RT	4	6.6E-2	9.7E-1	6.4E1
☐	SMART	UBQ	RT	4	8.7E-2	8.4E-1	6.7E1
☐	UP_SEQ_FEATURE	domain:Ubiquitin-like	RT	3	2.0E-1	1.0E0	9.7E1
☐	INTERPRO	Ubiquitin supergroup	RT	3	2.8E-1	1.0E0	9.9E1
	Annotation Cluster 37	Enrichment Score: 0.87	G	Count	P_Value	Benjamini	FDR
☐	SP_PIR_KEYWORDS	serine/threonine-protein kinase	RT	14	3.9E-2	4.3E-1	4.2E1
☐	GOTERM_MF_FAT	protein serine/threonine kinase activity	RT	15	4.7E-2	6.7E-1	5.0E1
☐	INTERPRO	Serine/threonine protein kinase-related	RT	13	6.3E-2	9.7E-1	6.2E1
☐	INTERPRO	Serine/threonine protein kinase, active site	RT	12	1.1E-1	9.9E-1	8.2E1
☐	INTERPRO	Protein kinase, ATP binding site	RT	14	1.3E-1	9.9E-1	8.8E1
☐	UP_SEQ_FEATURE	domain:Protein kinase	RT	14	1.4E-1	1.0E0	9.2E1
☐	GOTERM_MF_FAT	protein kinase activity	RT	17	1.5E-1	8.6E-1	9.0E1
☐	INTERPRO	Protein kinase, core	RT	14	1.8E-1	1.0E0	9.4E1
☐	UP_SEQ_FEATURE	binding site:ATP	RT	14	2.8E-1	1.0E0	1.0E2
☐	GOTERM_BP_FAT	protein amino acid phosphorylation	RT	17	3.2E-1	9.9E-1	1.0E2
☐	GOTERM_BP_FAT	phosphorylation	RT	18	5.0E-1	1.0E0	1.0E2
	Annotation Cluster 38	Enrichment Score: 0.84	G	Count	P_Value	Benjamini	FDR
☐	GOTERM_BP_FAT	stress-activated protein kinase signaling pathway	RT	5	4.3E-2	8.7E-1	5.2E1
☐	GOTERM_BP_FAT	JNK cascade	RT	4	1.3E-1	9.7E-1	8.9E1
☐	GOTERM_BP_FAT	MAPKKK cascade	RT	5	5.5E-1	1.0E0	1.0E2
	Annotation Cluster 39	Enrichment Score: 0.83	G	Count	P_Value	Benjamini	FDR
☐	KEGG_PATHWAY	Steroid biosynthesis	RT	3	3.5E-2	6.3E-1	3.3E1

Category	Term			Count	P_Value	Benjamini	FDR
Annotation Cluster 1	Enrichment Score: 6.89	G		Count	P_Value	Benjamini	FDR
SP_PIR_KEYWORDS	Steroid biosynthesis	RT		4	4.4E-2	4.5E-1	4.6E1
GOTERM_BP_FAT	cholesterol biosynthetic process	RT		3	1.0E-1	9.6E-1	8.4E1
GOTERM_BP_FAT	sterol biosynthetic process	RT		3	1.7E-1	9.8E-1	9.5E1
GOTERM_BP_FAT	steroid biosynthetic process	RT		4	2.6E-1	9.9E-1	9.9E1
GOTERM_BP_FAT	cholesterol metabolic process	RT		4	3.0E-1	9.9E-1	1.0E2
SP_PIR_KEYWORDS	lipid synthesis	RT		4	3.0E-1	8.6E-1	9.9E1
GOTERM_BP_FAT	sterol metabolic process	RT		4	3.5E-1	9.9E-1	1.0E2
Annotation Cluster 40	Enrichment Score: 0.83	G		Count	P_Value	Benjamini	FDR
GOTERM_CC_FAT	membrane-bounded vesicle	RT		17	7.1E-2	4.6E-1	6.3E1
GOTERM_CC_FAT	vesicle	RT		18	1.3E-1	6.3E-1	8.4E1
GOTERM_CC_FAT	cytoplasmic membrane-bounded vesicle	RT		15	1.6E-1	7.0E-1	9.0E1
GOTERM_CC_FAT	cytoplasmic vesicle	RT		15	3.3E-1	8.6E-1	9.9E1
Annotation Cluster 41	Enrichment Score: 0.81	G		Count	P_Value	Benjamini	FDR
BIOCARTA	Influence of Ras and Rho proteins on G1 to S Transition	RT		4	3.5E-2	8.8E-1	3.3E1
KEGG_PATHWAY	B cell receptor signaling pathway	RT		5	4.2E-2	6.2E-1	3.8E1
BIOCARTA	Angiotensin II mediated activation of JNK Pathway via Pyk2 dependent signaling	RT		4	4.7E-2	8.5E-1	4.2E1
BIOCARTA	Role of MAL in Rho-Mediated Activation of SRF	RT		3	1.1E-1	9.4E-1	7.5E1
BIOCARTA	Ras Signaling Pathway	RT		3	1.2E-1	9.3E-1	7.8E1
KEGG_PATHWAY	Renal cell carcinoma	RT		4	1.2E-1	6.1E-1	7.8E1
KEGG_PATHWAY	VEGF signaling pathway	RT		4	1.4E-1	5.8E-1	8.3E1
BIOCARTA	fMLP induced chemokine gene expression in HMC-1 cells	RT		3	1.8E-1	9.5E-1	8.9E1
BIOCARTA	BCR Signaling Pathway	RT		3	1.9E-1	9.4E-1	9.1E1
BIOCARTA	Links between Pyk2 and Map Kinases	RT		3	1.9E-1	9.4E-1	9.1E1
BIOCARTA	T Cell Receptor Signaling Pathway	RT		3	2.8E-1	9.6E-1	9.8E1
KEGG_PATHWAY	Fc epsilon RI signaling pathway	RT		3	4.0E-1	7.9E-1	1.0E2
KEGG_PATHWAY	Natural killer cell mediated cytotoxicity	RT		4	4.1E-1	8.0E-1	1.0E2
SP_PIR_KEYWORDS	Proto-oncogene	RT		4	8.3E-1	1.0E0	1.0E2
Annotation Cluster 42	Enrichment Score: 0.81	G		Count	P_Value	Benjamini	FDR
GOTERM_BP_FAT	positive regulation of protein polymerization	RT		4	1.5E-2	8.4E-1	2.2E1
GOTERM_BP_FAT	positive regulation of actin filament polymerization	RT		3	2.5E-2	8.5E-1	3.5E1
GOTERM_BP_FAT	positive regulation of protein complex assembly	RT		4	3.6E-2	8.8E-1	4.6E1
GOTERM_BP_FAT	positive regulation of cytoskeleton organization	RT		4	6.8E-2	9.3E-1	6.9E1
GOTERM_BP_FAT	positive regulation of organelle organization	RT		5	9.6E-2	9.6E-1	8.2E1
GOTERM_BP_FAT	regulation of protein complex assembly	RT		5	1.2E-1	9.7E-1	8.8E1
GOTERM_BP_FAT	regulation of protein polymerization	RT		4	1.7E-1	9.8E-1	9.6E1
GOTERM_BP_FAT	regulation of actin filament polymerization	RT		3	3.1E-1	9.9E-1	1.0E2
GOTERM_BP_FAT	regulation of cellular component biogenesis	RT		5	3.5E-1	9.9E-1	1.0E2
GOTERM_BP_FAT	regulation of actin polymerization or depolymerization	RT		3	3.7E-1	9.9E-1	1.0E2
GOTERM_BP_FAT	regulation of actin filament length	RT		3	3.8E-1	9.9E-1	1.0E2
GOTERM_BP_FAT	regulation of cytoskeleton organization	RT		4	5.4E-1	1.0E0	1.0E2
GOTERM_BP_FAT	regulation of actin cytoskeleton organization	RT		3	5.6E-1	1.0E0	1.0E2
GOTERM_BP_FAT	regulation of actin filament-based process	RT		3	5.8E-1	1.0E0	1.0E2
Annotation Cluster 43	Enrichment Score: 0.76	G		Count	P_Value	Benjamini	FDR
GOTERM_BP_FAT	protein localization	RT		24	1.6E-1	9.8E-1	9.5E1
GOTERM_BP_FAT	protein transport	RT		21	1.7E-1	9.8E-1	9.6E1
GOTERM_BP_FAT	establishment of protein localization	RT		21	1.9E-1	9.8E-1	9.7E1
Annotation Cluster 44	Enrichment Score: 0.73	G		Count	P_Value	Benjamini	FDR
GOTERM_BP_FAT	nucleic acid transport	RT		5	1.5E-1	9.8E-1	9.3E1
GOTERM_BP_FAT	RNA transport	RT		5	1.5E-1	9.8E-1	9.3E1
GOTERM_BP_FAT	establishment of RNA localization	RT		5	1.5E-1	9.8E-1	9.3E1

	Category	Term		Count	P_Value	Benjamini	FDR
	Annotation Cluster 1	Enrichment Score: 6.89	G	Count	P_Value	Benjamini	FDR
	GOTERM_BP_FAT	RNA localization	RT	5	1.6E-1	9.8E-1	9.4E1
	GOTERM_CC_FAT	nuclear pore	RT	4	1.9E-1	7.3E-1	9.4E1
	GOTERM_BP_FAT	nucleobase, nucleoside, nucleotide and nucleic acid transport	RT	5	2.1E-1	9.8E-1	9.8E1
	GOTERM_CC_FAT	pore complex	RT	4	2.7E-1	8.1E-1	9.8E1
	GOTERM_BP_FAT	mRNA transport	RT	4	2.7E-1	9.9E-1	1.0E2
	Annotation Cluster 45	Enrichment Score: 0.71	G	Count	P_Value	Benjamini	FDR
	GOTERM_BP_FAT	positive regulation of neurogenesis	RT	5	3.5E-2	9.0E-1	4.5E1
	GOTERM_BP_FAT	positive regulation of cell development	RT	5	5.6E-2	9.1E-1	6.2E1
	GOTERM_BP_FAT	regulation of cell development	RT	7	2.6E-1	9.9E-1	9.9E1
	GOTERM_BP_FAT	regulation of neurogenesis	RT	6	2.7E-1	9.9E-1	9.9E1
	GOTERM_BP_FAT	positive regulation of developmental process	RT	8	3.7E-1	9.9E-1	1.0E2
	GOTERM_BP_FAT	regulation of nervous system development	RT	6	3.8E-1	9.9E-1	1.0E2
	GOTERM_BP_FAT	positive regulation of cell differentiation	RT	6	5.3E-1	1.0E0	1.0E2
	Annotation Cluster 46	Enrichment Score: 0.69	G	Count	P_Value	Benjamini	FDR
	GOTERM_BP_FAT	regulation of phosphorylation	RT	14	1.8E-1	9.8E-1	9.6E1
	GOTERM_BP_FAT	regulation of phosphorus metabolic process	RT	14	2.2E-1	9.8E-1	9.8E1
	GOTERM_BP_FAT	regulation of phosphate metabolic process	RT	14	2.2E-1	9.8E-1	9.8E1
	Annotation Cluster 47	Enrichment Score: 0.68	G	Count	P_Value	Benjamini	FDR
	GOTERM_MF_FAT	protein serine/threonine phosphatase activity	RT	4	5.9E-2	7.0E-1	5.8E1
	GOTERM_BP_FAT	dephosphorylation	RT	6	2.2E-1	9.8E-1	9.8E1
	GOTERM_MF_FAT	phosphoprotein phosphatase activity	RT	6	2.3E-1	9.4E-1	9.8E1
	SP_PIR_KEYWORDS	protein phosphatase	RT	5	2.5E-1	8.1E-1	9.8E1
	GOTERM_BP_FAT	protein amino acid dephosphorylation	RT	5	3.0E-1	9.9E-1	1.0E2
	GOTERM_MF_FAT	phosphatase activity	RT	7	3.7E-1	9.8E-1	1.0E2
	Annotation Cluster 48	Enrichment Score: 0.66	G	Count	P_Value	Benjamini	FDR
	UP_SEQ_FEATURE	region of interest:Modulating	RT	3	3.0E-2	9.2E-1	3.9E1
	UP_SEQ_FEATURE	DNA-binding region:Nuclear receptor	RT	3	2.2E-1	1.0E0	9.8E1
	UP_SEQ_FEATURE	zinc finger region:NR C4-type	RT	3	2.2E-1	1.0E0	9.8E1
	INTERPRO	Zinc finger, nuclear hormone receptor-type	RT	3	2.4E-1	1.0E0	9.8E1
	INTERPRO	Steroid hormone receptor	RT	3	2.5E-1	1.0E0	9.9E1
	GOTERM_MF_FAT	steroid hormone receptor activity	RT	3	2.5E-1	9.5E-1	9.8E1
	INTERPRO	Nuclear hormone receptor, ligand-binding	RT	3	2.5E-1	1.0E0	9.9E1
	INTERPRO	Nuclear hormone receptor, ligand-binding, core	RT	3	2.5E-1	1.0E0	9.9E1
	INTERPRO	Zinc finger, NHR/GATA-type	RT	3	2.8E-1	1.0E0	9.9E1
	SMART	ZnF_C4	RT	3	2.8E-1	9.7E-1	9.8E1
	SMART	HOLI	RT	3	3.0E-1	9.7E-1	9.9E1
	GOTERM_MF_FAT	ligand-dependent nuclear receptor activity	RT	3	3.2E-1	9.7E-1	1.0E2
	Annotation Cluster 49	Enrichment Score: 0.64	G	Count	P_Value	Benjamini	FDR
	INTERPRO	Ubiquitin-conjugating enzyme, E2	RT	3	2.0E-1	1.0E0	9.7E1
	SMART	UBCc	RT	3	2.4E-1	9.6E-1	9.6E1
	INTERPRO	Ubiquitin-conjugating enzyme/RWD-like	RT	3	2.5E-1	1.0E0	9.9E1
	Annotation Cluster 50	Enrichment Score: 0.6	G	Count	P_Value	Benjamini	FDR
	GOTERM_BP_FAT	RNA splicing, via transesterification reactions with bulged adenosine as nucleophile	RT	6	2.2E-1	9.8E-1	9.8E1
	GOTERM_BP_FAT	RNA splicing, via transesterification reactions	RT	6	2.2E-1	9.8E-1	9.8E1
	GOTERM_BP_FAT	nuclear mRNA splicing, via spliceosome	RT	6	2.2E-1	9.8E-1	9.8E1
	GOTERM_BP_FAT	RNA splicing	RT	8	3.8E-1	9.9E-1	1.0E2
	Annotation Cluster 51	Enrichment Score: 0.59	G	Count	P_Value	Benjamini	FDR
	GOTERM_BP_FAT	positive regulation of DNA binding	RT	4	1.8E-1	9.8E-1	9.6E1
	GOTERM_BP_FAT	positive regulation of NF-kappaB transcription factor activity	RT	3	2.1E-1	9.9E-1	9.8E1
	GOTERM_BP_FAT	positive regulation of binding	RT	4	2.2E-1	9.9E-1	9.9E1

						Count	P_Value	Benjamini	FDR
	Annotation Cluster 1	Enrichment Score: 6.89	G			Count	P_Value	Benjamini	FDR
☐	GOTERM_BP_FAT	regulation of DNA binding	RT			5	2.5E-1	9.9E-1	9.9E1
☐	GOTERM_BP_FAT	positive regulation of transcription factor activity	RT			3	3.6E-1	9.9E-1	1.0E2
☐	GOTERM_BP_FAT	regulation of binding	RT			5	4.0E-1	9.9E-1	1.0E2
	Annotation Cluster 52	Enrichment Score: 0.58	G			Count	P_Value	Benjamini	FDR
☐	SP_PIR_KEYWORDS	mrna processing	RT			9	1.4E-1	7.2E-1	8.8E1
☐	GOTERM_BP_FAT	mRNA processing	RT			10	2.3E-1	9.8E-1	9.9E1
☐	GOTERM_BP_FAT	mRNA metabolic process	RT			10	3.7E-1	9.9E-1	1.0E2
☐	GOTERM_BP_FAT	RNA splicing	RT			8	3.8E-1	9.9E-1	1.0E2
	Annotation Cluster 53	Enrichment Score: 0.57	G			Count	P_Value	Benjamini	FDR
☐	GOTERM_BP_FAT	induction of apoptosis	RT			10	2.3E-1	9.9E-1	9.9E1
☐	GOTERM_BP_FAT	induction of programmed cell death	RT			10	2.3E-1	9.8E-1	9.9E1
☐	GOTERM_BP_FAT	positive regulation of apoptosis	RT			12	2.9E-1	9.9E-1	1.0E2
☐	GOTERM_BP_FAT	positive regulation of programmed cell death	RT			12	3.0E-1	9.9E-1	1.0E2
☐	GOTERM_BP_FAT	positive regulation of cell death	RT			12	3.0E-1	9.9E-1	1.0E2
	Annotation Cluster 54	Enrichment Score: 0.54	G			Count	P_Value	Benjamini	FDR
☐	PIR_SUPERFAMILY	PIRSF037037:Kelch-like_protein_gigaxonin	RT			3	1.6E-1	1.0E0	8.7E1
☐	UP_SEQ_FEATURE	repeat:Kelch 4	RT			4	1.6E-1	1.0E0	9.4E1
☐	SP_PIR_KEYWORDS	kelch repeat	RT			4	1.6E-1	7.5E-1	9.2E1
☐	INTERPRO	Kelch repeat type 1	RT			4	1.6E-1	1.0E0	9.3E1
☐	INTERPRO	Kelch-type beta propeller	RT			4	1.6E-1	1.0E0	9.3E1
☐	UP_SEQ_FEATURE	repeat:Kelch 3	RT			4	1.7E-1	1.0E0	9.5E1
☐	UP_SEQ_FEATURE	repeat:Kelch 1	RT			4	1.7E-1	1.0E0	9.5E1
☐	UP_SEQ_FEATURE	repeat:Kelch 2	RT			4	1.7E-1	1.0E0	9.5E1
☐	INTERPRO	Kelch-like protein, gigaxonin	RT			3	2.0E-1	1.0E0	9.7E1
☐	SMART	Kelch	RT			4	2.1E-1	9.7E-1	9.4E1
☐	UP_SEQ_FEATURE	repeat:Kelch 6	RT			3	2.8E-1	1.0E0	9.9E1
☐	INTERPRO	BTB/Kelch-associated	RT			3	3.1E-1	1.0E0	1.0E2
☐	UP_SEQ_FEATURE	repeat:Kelch 5	RT			3	3.8E-1	1.0E0	1.0E2
☐	INTERPRO	BTB/POZ	RT			4	4.8E-1	1.0E0	1.0E2
☐	INTERPRO	Kelch related	RT			3	4.8E-1	1.0E0	1.0E2
☐	INTERPRO	BTB/POZ-like	RT			4	7.1E-1	1.0E0	1.0E2
☐	INTERPRO	BTB/POZ fold	RT			4	7.1E-1	1.0E0	1.0E2
☐	UP_SEQ_FEATURE	domain:BTB	RT			3	7.8E-1	1.0E0	1.0E2
☐	SMART	BTB	RT			4	7.8E-1	1.0E0	1.0E2
	Annotation Cluster 55	Enrichment Score: 0.5	G			Count	P_Value	Benjamini	FDR
☐	GOTERM_BP_FAT	positive regulation of cell migration	RT			4	2.9E-1	9.9E-1	1.0E2
☐	GOTERM_BP_FAT	positive regulation of cell motion	RT			4	3.4E-1	9.9E-1	1.0E2
☐	GOTERM_BP_FAT	positive regulation of locomotion	RT			4	3.4E-1	9.9E-1	1.0E2
	Annotation Cluster 56	Enrichment Score: 0.49	G			Count	P_Value	Benjamini	FDR
☐	INTERPRO	Zinc finger, PHD-finger	RT			4	2.5E-1	1.0E0	9.9E1
☐	INTERPRO	Zinc finger, PHD-type	RT			4	2.8E-1	1.0E0	9.9E1
☐	UP_SEQ_FEATURE	zinc finger region:PHD-type	RT			3	2.8E-1	1.0E0	9.9E1
☐	SMART	PHD	RT			4	3.4E-1	9.8E-1	9.9E1
☐	INTERPRO	Zinc finger, PHD-type, conserved site	RT			3	5.7E-1	1.0E0	1.0E2
	Annotation Cluster 57	Enrichment Score: 0.47	G			Count	P_Value	Benjamini	FDR
☐	KEGG_PATHWAY	GnRH signaling pathway	RT			4	2.5E-1	6.9E-1	9.6E1
☐	KEGG_PATHWAY	Glioma	RT			3	3.0E-1	7.3E-1	9.8E1
☐	KEGG_PATHWAY	Melanogenesis	RT			3	5.2E-1	8.6E-1	1.0E2
	Annotation Cluster 58	Enrichment Score: 0.47	G			Count	P_Value	Benjamini	FDR
☐	INTERPRO	Zinc finger, C2H2-like	RT			20	2.7E-1	1.0E0	9.9E1
☐	INTERPRO	Zinc finger, C2H2-type	RT			19	3.3E-1	1.0E0	1.0E2
☐	SMART	ZnF_C2H2	RT			20	4.5E-1	9.9E-1	1.0E2
	Annotation Cluster 59	Enrichment Score: 0.47	G			Count	P_Value	Benjamini	FDR

	Category	Term			Count	P_Value	Benjamini	FDR
Annotation Cluster 1		Enrichment Score: 6.89	G		Count	P_Value	Benjamini	FDR
	UP_SEQ_FEATURE	short sequence motif:Effector region	RT		4	2.9E-1	1.0E0	1.0E2
	INTERPRO	Ras GTPase	RT		5	2.9E-1	1.0E0	9.9E1
	INTERPRO	Ras	RT		4	4.7E-1	1.0E0	1.0E2
Annotation Cluster 60		Enrichment Score: 0.46	G		Count	P_Value	Benjamini	FDR
	GOTERM_BP_FAT	regulation of cell migration	RT		6	2.8E-1	9.9E-1	1.0E2
	GOTERM_BP_FAT	regulation of locomotion	RT		6	3.8E-1	9.9E-1	1.0E2
	GOTERM_BP_FAT	regulation of cell motion	RT		6	3.8E-1	9.9E-1	1.0E2
Annotation Cluster 61		Enrichment Score: 0.45	G		Count	P_Value	Benjamini	FDR
	GOTERM_BP_FAT	phosphate metabolic process	RT		24	3.0E-1	9.9E-1	1.0E2
	GOTERM_BP_FAT	phosphorus metabolic process	RT		24	3.0E-1	9.9E-1	1.0E2
	GOTERM_BP_FAT	phosphorylation	RT		18	5.0E-1	1.0E0	1.0E2
Annotation Cluster 62		Enrichment Score: 0.43	G		Count	P_Value	Benjamini	FDR
	KEGG_PATHWAY	Homologous recombination	RT		3	8.5E-2	6.7E-1	6.4E1
	GOTERM_BP_FAT	M phase of meiotic cell cycle	RT		3	6.1E-1	1.0E0	1.0E2
	GOTERM_BP_FAT	meiosis	RT		3	6.1E-1	1.0E0	1.0E2
	GOTERM_BP_FAT	meiotic cell cycle	RT		3	6.2E-1	1.0E0	1.0E2
Annotation Cluster 63		Enrichment Score: 0.42	G		Count	P_Value	Benjamini	FDR
	SP_PIR_KEYWORDS	dioxygenase	RT		3	3.7E-1	9.0E-1	1.0E2
	GOTERM_MF_FAT	oxidoreductase activity, acting on single donors with incorporation of molecular oxygen, incorporation of two atoms of oxygen	RT		3	3.8E-1	9.8E-1	1.0E2
	GOTERM_MF_FAT	oxidoreductase activity, acting on single donors with incorporation of molecular oxygen	RT		3	3.8E-1	9.8E-1	1.0E2
Annotation Cluster 64		Enrichment Score: 0.42	G		Count	P_Value	Benjamini	FDR
	GOTERM_BP_FAT	regulation of kinase activity	RT		10	3.3E-1	9.9E-1	1.0E2
	GOTERM_BP_FAT	regulation of transferase activity	RT		10	3.8E-1	9.9E-1	1.0E2
	GOTERM_BP_FAT	regulation of protein kinase activity	RT		9	4.4E-1	9.9E-1	1.0E2
Annotation Cluster 65		Enrichment Score: 0.41	G		Count	P_Value	Benjamini	FDR
	UP_SEQ_FEATURE	nucleotide phosphate-binding region:ATP	RT		25	1.4E-1	1.0E0	9.2E1
	SP_PIR_KEYWORDS	atp-binding	RT		30	3.0E-1	8.6E-1	9.9E1
	GOTERM_MF_FAT	ATP binding	RT		32	3.9E-1	9.8E-1	1.0E2
	GOTERM_MF_FAT	adenyl ribonucleotide binding	RT		32	4.2E-1	9.8E-1	1.0E2
	GOTERM_MF_FAT	adenyl nucleotide binding	RT		32	5.5E-1	9.9E-1	1.0E2
	GOTERM_MF_FAT	purine nucleoside binding	RT		32	5.8E-1	1.0E0	1.0E2
	GOTERM_MF_FAT	nucleoside binding	RT		32	6.0E-1	1.0E0	1.0E2
Annotation Cluster 66		Enrichment Score: 0.38	G		Count	P_Value	Benjamini	FDR
	GOTERM_BP_FAT	regulation of nuclear division	RT		3	3.3E-1	9.9E-1	1.0E2
	GOTERM_BP_FAT	regulation of mitosis	RT		3	3.3E-1	9.9E-1	1.0E2
	GOTERM_BP_FAT	regulation of cell cycle process	RT		3	6.9E-1	1.0E0	1.0E2
Annotation Cluster 67		Enrichment Score: 0.37	G		Count	P_Value	Benjamini	FDR
	KEGG_PATHWAY	Fructose and mannose metabolism	RT		3	1.2E-1	6.7E-1	7.6E1
	SP_PIR_KEYWORDS	glycolysis	RT		3	2.1E-1	7.8E-1	9.6E1
	GOTERM_BP_FAT	glycolysis	RT		3	2.6E-1	9.9E-1	9.9E1
	KEGG_PATHWAY	Glycolysis / Gluconeogenesis	RT		3	2.8E-1	7.1E-1	9.8E1
	GOTERM_BP_FAT	glucose catabolic process	RT		3	3.4E-1	9.9E-1	1.0E2
	GOTERM_BP_FAT	hexose catabolic process	RT		3	4.3E-1	9.9E-1	1.0E2
	GOTERM_BP_FAT	monosaccharide catabolic process	RT		3	4.4E-1	9.9E-1	1.0E2
	GOTERM_BP_FAT	alcohol catabolic process	RT		3	5.1E-1	1.0E0	1.0E2
	GOTERM_BP_FAT	cellular carbohydrate catabolic process	RT		3	5.3E-1	1.0E0	1.0E2
	GOTERM_BP_FAT	glucose metabolic process	RT		4	6.2E-1	1.0E0	1.0E2
	GOTERM_BP_FAT	carbohydrate catabolic process	RT		3	6.7E-1	1.0E0	1.0E2
	GOTERM_BP_FAT	monosaccharide metabolic process	RT		5	6.9E-1	1.0E0	1.0E2
	GOTERM_BP_FAT	hexose metabolic process	RT		4	7.7E-1	1.0E0	1.0E2
	GOTERM_BP_FAT	generation of precursor metabolites and energy	RT		5	9.0E-1	1.0E0	1.0E2
Annotation Cluster 68		Enrichment Score: 0.35	G		Count	P_Value	Benjamini	FDR

	Category	Term		Count	P_Value	Benjamini	FDR
	Annotation Cluster 1	Enrichment Score: 6.89	G	Count	P_Value	Benjamini	FDR
☐	GOTERM_BP_FAT	regulation of intracellular transport	RT	4	1.9E-1	9.8E-1	9.7E1
☐	GOTERM_BP_FAT	regulation of intracellular protein transport	RT	3	3.0E-1	9.9E-1	1.0E2
☐	GOTERM_BP_FAT	regulation of nucleocytoplasmic transport	RT	3	3.4E-1	9.9E-1	1.0E2
☐	GOTERM_BP_FAT	regulation of protein transport	RT	3	6.9E-1	1.0E0	1.0E2
☐	GOTERM_BP_FAT	regulation of establishment of protein localization	RT	3	7.2E-1	1.0E0	1.0E2
☐	GOTERM_BP_FAT	regulation of protein localization	RT	3	7.9E-1	1.0E0	1.0E2
	Annotation Cluster 69	Enrichment Score: 0.35	G	Count	P_Value	Benjamini	FDR
☐	GOTERM_BP_FAT	regulation of cellular response to stress	RT	4	3.7E-1	9.9E-1	1.0E2
☐	GOTERM_BP_FAT	regulation of JNK cascade	RT	3	4.0E-1	9.9E-1	1.0E2
☐	GOTERM_BP_FAT	regulation of stress-activated protein kinase signaling pathway	RT	3	4.3E-1	9.9E-1	1.0E2
☐	GOTERM_BP_FAT	regulation of MAPKKK cascade	RT	3	6.7E-1	1.0E0	1.0E2
	Annotation Cluster 70	Enrichment Score: 0.34	G	Count	P_Value	Benjamini	FDR
☐	INTERPRO	Peptidase C19, ubiquitin carboxyl-terminal hydrolase 2	RT	3	3.5E-1	1.0E0	1.0E2
☐	GOTERM_MF_FAT	ubiquitin thiolesterase activity	RT	3	4.4E-1	9.9E-1	1.0E2
☐	GOTERM_MF_FAT	thiolester hydrolase activity	RT	3	6.0E-1	1.0E0	1.0E2
	Annotation Cluster 71	Enrichment Score: 0.34	G	Count	P_Value	Benjamini	FDR
☐	INTERPRO	RNA recognition motif, RNP-1	RT	6	4.3E-1	1.0E0	1.0E2
☐	INTERPRO	Nucleotide-binding, alpha-beta plait	RT	6	4.3E-1	1.0E0	1.0E2
☐	SMART	RRM	RT	6	5.3E-1	1.0E0	1.0E2
	Annotation Cluster 72	Enrichment Score: 0.33	G	Count	P_Value	Benjamini	FDR
☐	GOTERM_BP_FAT	macromolecular complex assembly	RT	16	4.2E-1	9.9E-1	1.0E2
☐	GOTERM_BP_FAT	protein complex biogenesis	RT	12	4.9E-1	1.0E0	1.0E2
☐	GOTERM_BP_FAT	protein complex assembly	RT	12	4.9E-1	1.0E0	1.0E2
	Annotation Cluster 73	Enrichment Score: 0.33	G	Count	P_Value	Benjamini	FDR
☐	GOTERM_BP_FAT	negative regulation of nucleobase, nucleoside, nucleotide and nucleic acid metabolic process	RT	13	3.9E-1	9.9E-1	1.0E2
☐	GOTERM_BP_FAT	negative regulation of nitrogen compound metabolic process	RT	13	4.0E-1	9.9E-1	1.0E2
☐	GOTERM_BP_FAT	negative regulation of macromolecule biosynthetic process	RT	13	4.8E-1	1.0E0	1.0E2
☐	GOTERM_BP_FAT	negative regulation of gene expression	RT	12	4.9E-1	1.0E0	1.0E2
☐	GOTERM_BP_FAT	negative regulation of transcription	RT	11	5.0E-1	1.0E0	1.0E2
☐	GOTERM_BP_FAT	negative regulation of cellular biosynthetic process	RT	13	5.0E-1	1.0E0	1.0E2
☐	GOTERM_BP_FAT	negative regulation of biosynthetic process	RT	13	5.4E-1	1.0E0	1.0E2
	Annotation Cluster 74	Enrichment Score: 0.3	G	Count	P_Value	Benjamini	FDR
☐	GOTERM_BP_FAT	immune response-activating signal transduction	RT	3	3.0E-1	9.9E-1	1.0E2
☐	GOTERM_BP_FAT	activation of immune response	RT	4	3.1E-1	9.9E-1	1.0E2
☐	GOTERM_BP_FAT	immune response-regulating signal transduction	RT	3	3.3E-1	9.9E-1	1.0E2
☐	GOTERM_BP_FAT	positive regulation of immune response	RT	4	5.9E-1	1.0E0	1.0E2
☐	GOTERM_BP_FAT	positive regulation of response to stimulus	RT	5	7.3E-1	1.0E0	1.0E2
☐	GOTERM_BP_FAT	positive regulation of immune system process	RT	5	7.4E-1	1.0E0	1.0E2
☐	GOTERM_BP_FAT	immune effector process	RT	3	7.7E-1	1.0E0	1.0E2
	Annotation Cluster 75	Enrichment Score: 0.3	G	Count	P_Value	Benjamini	FDR
☐	INTERPRO	Tetratricopeptide repeat	RT	4	4.2E-1	1.0E0	1.0E2
☐	INTERPRO	Tetratricopeptide region	RT	4	4.5E-1	1.0E0	1.0E2
☐	SMART	TPR	RT	4	5.0E-1	1.0E0	1.0E2
☐	UP_SEQ_FEATURE	repeat:TPR 3	RT	4	5.2E-1	1.0E0	1.0E2
☐	UP_SEQ_FEATURE	repeat:TPR 2	RT	4	5.8E-1	1.0E0	1.0E2
☐	UP_SEQ_FEATURE	repeat:TPR 1	RT	4	5.8E-1	1.0E0	1.0E2
	Annotation Cluster 76	Enrichment Score: 0.3	G	Count	P_Value	Benjamini	FDR
☐	GOTERM_BP_FAT	cellular protein localization	RT	10	4.9E-1	1.0E0	1.0E2
☐	GOTERM_BP_FAT	cellular macromolecule localization	RT	10	4.9E-1	1.0E0	1.0E2
☐	GOTERM_BP_FAT	intracellular protein transport	RT	9	5.3E-1	1.0E0	1.0E2

					Count	P_Value	Benjamini	FDR
	Annotation Cluster 1	Enrichment Score: 6.89	G		Count	P_Value	Benjamini	FDR
	Annotation Cluster 77	Enrichment Score: 0.29	G		Count	P_Value	Benjamini	FDR
☐	GOTERM_BP_FAT	regulation of caspase activity	RT		3	4.9E-1	1.0E0	1.0E2
☐	GOTERM_BP_FAT	regulation of endopeptidase activity	RT		3	5.1E-1	1.0E0	1.0E2
☐	GOTERM_BP_FAT	regulation of peptidase activity	RT		3	5.4E-1	1.0E0	1.0E2
	Annotation Cluster 78	Enrichment Score: 0.28	G		Count	P_Value	Benjamini	FDR
☐	GOTERM_BP_FAT	negative regulation of RNA metabolic process	RT		9	4.9E-1	1.0E0	1.0E2
☐	GOTERM_BP_FAT	negative regulation of transcription from RNA polymerase II promoter	RT		7	4.9E-1	1.0E0	1.0E2
☐	GOTERM_BP_FAT	negative regulation of transcription, DNA-dependent	RT		8	6.2E-1	1.0E0	1.0E2
	Annotation Cluster 79	Enrichment Score: 0.27	G		Count	P_Value	Benjamini	FDR
☐	GOTERM_BP_FAT	in utero embryonic development	RT		6	3.1E-1	9.9E-1	1.0E2
☐	GOTERM_BP_FAT	chordate embryonic development	RT		7	7.0E-1	1.0E0	1.0E2
☐	GOTERM_BP_FAT	embryonic development ending in birth or egg hatching	RT		7	7.0E-1	1.0E0	1.0E2
	Annotation Cluster 80	Enrichment Score: 0.27	G		Count	P_Value	Benjamini	FDR
☐	GOTERM_BP_FAT	positive regulation of defense response	RT		3	4.5E-1	9.9E-1	1.0E2
☐	GOTERM_BP_FAT	regulation of cytokine biosynthetic process	RT		3	4.6E-1	9.9E-1	1.0E2
☐	GOTERM_BP_FAT	regulation of cytokine production	RT		4	7.3E-1	1.0E0	1.0E2
	Annotation Cluster 81	Enrichment Score: 0.27	G		Count	P_Value	Benjamini	FDR
☐	GOTERM_BP_FAT	limb morphogenesis	RT		4	3.4E-1	9.9E-1	1.0E2
☐	GOTERM_BP_FAT	appendage morphogenesis	RT		4	3.4E-1	9.9E-1	1.0E2
☐	GOTERM_BP_FAT	appendage development	RT		4	3.7E-1	9.9E-1	1.0E2
☐	GOTERM_BP_FAT	limb development	RT		4	3.7E-1	9.9E-1	1.0E2
☐	GOTERM_BP_FAT	embryonic appendage morphogenesis	RT		3	5.5E-1	1.0E0	1.0E2
☐	GOTERM_BP_FAT	embryonic limb morphogenesis	RT		3	5.5E-1	1.0E0	1.0E2
☐	GOTERM_BP_FAT	lung development	RT		3	6.2E-1	1.0E0	1.0E2
☐	GOTERM_BP_FAT	respiratory tube development	RT		3	6.3E-1	1.0E0	1.0E2
☐	GOTERM_BP_FAT	respiratory system development	RT		3	6.6E-1	1.0E0	1.0E2
☐	GOTERM_BP_FAT	tube development	RT		3	9.5E-1	1.0E0	1.0E2
☐	GOTERM_BP_FAT	embryonic morphogenesis	RT		4	9.6E-1	1.0E0	1.0E2
	Annotation Cluster 82	Enrichment Score: 0.26	G		Count	P_Value	Benjamini	FDR
☐	UP_SEQ_FEATURE	repeat:LRR 4	RT		7	3.8E-1	1.0E0	1.0E2
☐	UP_SEQ_FEATURE	repeat:LRR 3	RT		7	5.5E-1	1.0E0	1.0E2
☐	UP_SEQ_FEATURE	repeat:LRR 1	RT		7	6.1E-1	1.0E0	1.0E2
☐	UP_SEQ_FEATURE	repeat:LRR 2	RT		7	6.1E-1	1.0E0	1.0E2
☐	SP_PIR_KEYWORDS	leucine-rich repeat	RT		7	6.1E-1	9.7E-1	1.0E2
	Annotation Cluster 83	Enrichment Score: 0.25	G		Count	P_Value	Benjamini	FDR
☐	GOTERM_BP_FAT	nuclear import	RT		4	2.8E-1	9.9E-1	1.0E2
☐	GOTERM_BP_FAT	protein import into nucleus	RT		3	5.4E-1	1.0E0	1.0E2
☐	GOTERM_BP_FAT	protein localization in nucleus	RT		3	5.9E-1	1.0E0	1.0E2
☐	GOTERM_BP_FAT	protein import	RT		3	7.6E-1	1.0E0	1.0E2
☐	GOTERM_BP_FAT	protein localization in organelle	RT		3	8.1E-1	1.0E0	1.0E2
	Annotation Cluster 84	Enrichment Score: 0.24	G		Count	P_Value	Benjamini	FDR
☐	KEGG_PATHWAY	Spliceosome	RT		4	3.8E-1	7.8E-1	1.0E2
☐	SP_PIR_KEYWORDS	Spliceosome	RT		3	6.9E-1	9.8E-1	1.0E2
☐	GOTERM_CC_FAT	spliceosome	RT		3	7.1E-1	9.9E-1	1.0E2
	Annotation Cluster 85	Enrichment Score: 0.23	G		Count	P_Value	Benjamini	FDR
☐	GOTERM_BP_FAT	cell projection morphogenesis	RT		7	4.1E-1	9.9E-1	1.0E2
☐	GOTERM_BP_FAT	cell part morphogenesis	RT		7	4.5E-1	9.9E-1	1.0E2
☐	GOTERM_BP_FAT	cell projection organization	RT		9	5.1E-1	1.0E0	1.0E2
☐	GOTERM_BP_FAT	cellular component morphogenesis	RT		9	5.9E-1	1.0E0	1.0E2
☐	GOTERM_BP_FAT	cell morphogenesis	RT		8	6.2E-1	1.0E0	1.0E2
☐	GOTERM_BP_FAT	neuron development	RT		6	8.4E-1	1.0E0	1.0E2
☐	GOTERM_BP_FAT	neuron differentiation	RT		7	9.0E-1	1.0E0	1.0E2
	Annotation Cluster 86	Enrichment Score: 0.22	G		Count	P_Value	Benjamini	FDR

Annotation Cluster 1		Enrichment Score: 6.89	G		Count	P_Value	Benjamini	FDR
	GOTERM_BP_FAT	cellular ion homeostasis	RT		9	5.3E-1	1.0E0	1.0E2
	GOTERM_BP_FAT	cellular chemical homeostasis	RT		9	5.5E-1	1.0E0	1.0E2
	GOTERM_BP_FAT	ion homeostasis	RT		9	6.3E-1	1.0E0	1.0E2
	GOTERM_BP_FAT	chemical homeostasis	RT		10	7.5E-1	1.0E0	1.0E2
Annotation Cluster 87		Enrichment Score: 0.22	G		Count	P_Value	Benjamini	FDR
	GOTERM_BP_FAT	purine ribonucleotide biosynthetic process	RT		4	4.4E-1	9.9E-1	1.0E2
	GOTERM_BP_FAT	ribonucleotide biosynthetic process	RT		4	4.8E-1	1.0E0	1.0E2
	GOTERM_BP_FAT	nucleoside triphosphate metabolic process	RT		4	5.2E-1	1.0E0	1.0E2
	GOTERM_BP_FAT	nucleotide biosynthetic process	RT		5	5.5E-1	1.0E0	1.0E2
	GOTERM_BP_FAT	purine ribonucleotide metabolic process	RT		4	5.5E-1	1.0E0	1.0E2
	GOTERM_BP_FAT	ATP biosynthetic process	RT		3	5.6E-1	1.0E0	1.0E2
	GOTERM_BP_FAT	nucleobase, nucleoside and nucleotide biosynthetic process	RT		5	5.8E-1	1.0E0	1.0E2
	GOTERM_BP_FAT	nucleobase, nucleoside, nucleotide and nucleic acid biosynthetic process	RT		5	5.8E-1	1.0E0	1.0E2
	GOTERM_BP_FAT	ribonucleotide metabolic process	RT		4	6.0E-1	1.0E0	1.0E2
	GOTERM_BP_FAT	purine nucleotide biosynthetic process	RT		4	6.0E-1	1.0E0	1.0E2
	GOTERM_BP_FAT	purine ribonucleoside triphosphate biosynthetic process	RT		3	6.1E-1	1.0E0	1.0E2
	GOTERM_BP_FAT	ribonucleoside triphosphate biosynthetic process	RT		3	6.2E-1	1.0E0	1.0E2
	GOTERM_BP_FAT	purine nucleoside triphosphate biosynthetic process	RT		3	6.2E-1	1.0E0	1.0E2
	GOTERM_BP_FAT	nucleoside triphosphate biosynthetic process	RT		3	6.3E-1	1.0E0	1.0E2
	GOTERM_BP_FAT	ATP metabolic process	RT		3	6.5E-1	1.0E0	1.0E2
	GOTERM_BP_FAT	purine ribonucleoside triphosphate metabolic process	RT		3	7.1E-1	1.0E0	1.0E2
	GOTERM_BP_FAT	ribonucleoside triphosphate metabolic process	RT		3	7.1E-1	1.0E0	1.0E2
	GOTERM_BP_FAT	purine nucleoside triphosphate metabolic process	RT		3	7.3E-1	1.0E0	1.0E2
	GOTERM_BP_FAT	purine nucleotide metabolic process	RT		4	7.5E-1	1.0E0	1.0E2
	GOTERM_BP_FAT	nitrogen compound biosynthetic process	RT		6	8.1E-1	1.0E0	1.0E2
Annotation Cluster 88		Enrichment Score: 0.2	G		Count	P_Value	Benjamini	FDR
	GOTERM_BP_FAT	leukocyte activation	RT		6	5.7E-1	1.0E0	1.0E2
	GOTERM_BP_FAT	lymphocyte activation	RT		5	6.0E-1	1.0E0	1.0E2
	GOTERM_BP_FAT	cell activation	RT		6	7.2E-1	1.0E0	1.0E2
Annotation Cluster 89		Enrichment Score: 0.16	G		Count	P_Value	Benjamini	FDR
	INTERPRO	EF-HAND 1	RT		5	6.5E-1	1.0E0	1.0E2
	UP_SEQ_FEATURE	calcium-binding region:2	RT		3	6.7E-1	1.0E0	1.0E2
	UP_SEQ_FEATURE	domain:EF-hand 2	RT		4	7.0E-1	1.0E0	1.0E2
	UP_SEQ_FEATURE	domain:EF-hand 1	RT		4	7.0E-1	1.0E0	1.0E2
	UP_SEQ_FEATURE	calcium-binding region:1	RT		3	7.2E-1	1.0E0	1.0E2
Annotation Cluster 90		Enrichment Score: 0.16	G		Count	P_Value	Benjamini	FDR
	GOTERM_CC_FAT	focal adhesion	RT		3	5.8E-1	9.7E-1	1.0E2
	GOTERM_CC_FAT	cell-substrate adherens junction	RT		3	6.0E-1	9.7E-1	1.0E2
	GOTERM_CC_FAT	cell-substrate junction	RT		3	6.3E-1	9.8E-1	1.0E2
	GOTERM_CC_FAT	basolateral plasma membrane	RT		4	7.4E-1	9.9E-1	1.0E2
	GOTERM_CC_FAT	adherens junction	RT		3	7.9E-1	9.9E-1	1.0E2
	GOTERM_CC_FAT	anchoring junction	RT		3	8.4E-1	1.0E0	1.0E2
Annotation Cluster 91		Enrichment Score: 0.16	G		Count	P_Value	Benjamini	FDR
	UP_SEQ_FEATURE	repeat:LRR 6	RT		5	6.0E-1	1.0E0	1.0E2
	UP_SEQ_FEATURE	repeat:LRR 5	RT		5	6.8E-1	1.0E0	1.0E2
	INTERPRO	Leucine-rich repeat	RT		4	8.1E-1	1.0E0	1.0E2
Annotation Cluster 92		Enrichment Score: 0.16	G		Count	P_Value	Benjamini	FDR
	GOTERM_BP_FAT	angiogenesis	RT		4	6.0E-1	1.0E0	1.0E2
	GOTERM_BP_FAT	blood vessel morphogenesis	RT		5	6.5E-1	1.0E0	1.0E2
	GOTERM_BP_FAT	blood vessel development	RT		5	7.6E-1	1.0E0	1.0E2
	GOTERM_BP_FAT	vasculature development	RT		5	7.7E-1	1.0E0	1.0E2

Category	Term		Count	P_Value	Benjamini	FDR
Annotation Cluster 1	Enrichment Score: 6.89	G	Count	P_Value	Benjamini	FDR
Annotation Cluster 93	Enrichment Score: 0.15	G	Count	P_Value	Benjamini	FDR
GOTERM_BP_FAT	axonogenesis	RT	5	5.8E-1	1.0E0	1.0E2
GOTERM_BP_FAT	cell morphogenesis involved in neuron differentiation	RT	5	6.4E-1	1.0E0	1.0E2
GOTERM_BP_FAT	neuron projection morphogenesis	RT	5	6.5E-1	1.0E0	1.0E2
GOTERM_BP_FAT	cell morphogenesis involved in differentiation	RT	5	7.5E-1	1.0E0	1.0E2
GOTERM_BP_FAT	neuron projection development	RT	5	7.9E-1	1.0E0	1.0E2
GOTERM_BP_FAT	neuron development	RT	6	8.4E-1	1.0E0	1.0E2
Annotation Cluster 94	Enrichment Score: 0.15	G	Count	P_Value	Benjamini	FDR
GOTERM_BP_FAT	taxis	RT	4	6.5E-1	1.0E0	1.0E2
GOTERM_BP_FAT	chemotaxis	RT	4	6.5E-1	1.0E0	1.0E2
GOTERM_BP_FAT	locomotory behavior	RT	5	8.3E-1	1.0E0	1.0E2
Annotation Cluster 95	Enrichment Score: 0.13	G	Count	P_Value	Benjamini	FDR
UP_SEQ_FEATURE	zinc finger region:C2H2-type 13	RT	5	5.7E-1	1.0E0	1.0E2
UP_SEQ_FEATURE	zinc finger region:C2H2-type 14	RT	4	6.1E-1	1.0E0	1.0E2
UP_SEQ_FEATURE	zinc finger region:C2H2-type 12	RT	5	7.4E-1	1.0E0	1.0E2
UP_SEQ_FEATURE	zinc finger region:C2H2-type 11	RT	5	8.4E-1	1.0E0	1.0E2
UP_SEQ_FEATURE	zinc finger region:C2H2-type 9	RT	6	8.7E-1	1.0E0	1.0E2
UP_SEQ_FEATURE	zinc finger region:C2H2-type 10	RT	5	9.0E-1	1.0E0	1.0E2
Annotation Cluster 96	Enrichment Score: 0.12	G	Count	P_Value	Benjamini	FDR
GOTERM_BP_FAT	regulation of Ras GTPase activity	RT	3	6.4E-1	1.0E0	1.0E2
GOTERM_BP_FAT	regulation of GTPase activity	RT	3	7.3E-1	1.0E0	1.0E2
GOTERM_MF_FAT	small GTPase regulator activity	RT	4	9.1E-1	1.0E0	1.0E2
Annotation Cluster 97	Enrichment Score: 0.12	G	Count	P_Value	Benjamini	FDR
GOTERM_BP_FAT	positive regulation of nitrogen compound metabolic process	RT	14	5.9E-1	1.0E0	1.0E2
GOTERM_BP_FAT	positive regulation of macromolecule biosynthetic process	RT	13	7.2E-1	1.0E0	1.0E2
GOTERM_BP_FAT	positive regulation of cellular biosynthetic process	RT	13	7.8E-1	1.0E0	1.0E2
GOTERM_BP_FAT	positive regulation of biosynthetic process	RT	13	7.9E-1	1.0E0	1.0E2
GOTERM_BP_FAT	positive regulation of transcription	RT	10	8.4E-1	1.0E0	1.0E2
GOTERM_BP_FAT	positive regulation of gene expression	RT	10	8.6E-1	1.0E0	1.0E2
Annotation Cluster 98	Enrichment Score: 0.12	G	Count	P_Value	Benjamini	FDR
UP_SEQ_FEATURE	domain:PDZ	RT	3	6.4E-1	1.0E0	1.0E2
INTERPRO	PDZ/DHR/GLGF	RT	3	8.1E-1	1.0E0	1.0E2
SMART	PDZ	RT	3	8.6E-1	1.0E0	1.0E2
Annotation Cluster 99	Enrichment Score: 0.11	G	Count	P_Value	Benjamini	FDR
GOTERM_BP_FAT	cellular di-, tri-valent inorganic cation homeostasis	RT	5	7.0E-1	1.0E0	1.0E2
GOTERM_BP_FAT	di-, tri-valent inorganic cation homeostasis	RT	5	7.4E-1	1.0E0	1.0E2
GOTERM_BP_FAT	cellular calcium ion homeostasis	RT	4	7.4E-1	1.0E0	1.0E2
GOTERM_BP_FAT	calcium ion homeostasis	RT	4	7.6E-1	1.0E0	1.0E2
GOTERM_BP_FAT	cellular metal ion homeostasis	RT	4	7.8E-1	1.0E0	1.0E2
GOTERM_BP_FAT	cellular cation homeostasis	RT	5	7.8E-1	1.0E0	1.0E2
GOTERM_BP_FAT	metal ion homeostasis	RT	4	8.1E-1	1.0E0	1.0E2
GOTERM_BP_FAT	cation homeostasis	RT	5	8.5E-1	1.0E0	1.0E2
Annotation Cluster 100	Enrichment Score: 0.11	G	Count	P_Value	Benjamini	FDR
UP_SEQ_FEATURE	zinc finger region:C2H2-type 4	RT	11	7.1E-1	1.0E0	1.0E2
UP_SEQ_FEATURE	zinc finger region:C2H2-type 5	RT	10	7.4E-1	1.0E0	1.0E2
UP_SEQ_FEATURE	zinc finger region:C2H2-type 2	RT	11	7.7E-1	1.0E0	1.0E2
UP_SEQ_FEATURE	zinc finger region:C2H2-type 3	RT	11	7.9E-1	1.0E0	1.0E2
INTERPRO	Zinc finger, C2H2-type/integrase, DNA-binding	RT	10	8.9E-1	1.0E0	1.0E2
Annotation Cluster 101	Enrichment Score: 0.1	G	Count	P_Value	Benjamini	FDR
GOTERM_BP_FAT	hemopoiesis	RT	5	7.3E-1	1.0E0	1.0E2
GOTERM_BP_FAT	hemopoietic or lymphoid organ development	RT	5	8.0E-1	1.0E0	1.0E2
GOTERM_BP_FAT	immune system development	RT	5	8.3E-1	1.0E0	1.0E2

Annotation Cluster 1	Enrichment Score: 6.89		Count	P_Value	Benjamini	FDR
Annotation Cluster 102	Enrichment Score: 0.1		Count	P_Value	Benjamini	FDR
GOTERM_BP_FAT	positive regulation of nucleobase, nucleoside, nucleotide and nucleic acid metabolic process	RT	13	6.6E-1	1.0E0	1.0E2
GOTERM_BP_FAT	positive regulation of transcription, DNA-dependent	RT	9	7.9E-1	1.0E0	1.0E2
GOTERM_BP_FAT	positive regulation of transcription from RNA polymerase II promoter	RT	7	7.9E-1	1.0E0	1.0E2
GOTERM_BP_FAT	positive regulation of RNA metabolic process	RT	9	7.9E-1	1.0E0	1.0E2
GOTERM_BP_FAT	positive regulation of transcription	RT	10	8.4E-1	1.0E0	1.0E2
GOTERM_BP_FAT	positive regulation of gene expression	RT	10	8.6E-1	1.0E0	1.0E2
Annotation Cluster 103	Enrichment Score: 0.1		Count	P_Value	Benjamini	FDR
INTERPRO	Leucine-rich repeat, typical subtype	RT	3	7.5E-1	1.0E0	1.0E2
SMART	LRR_TYP	RT	3	8.0E-1	1.0E0	1.0E2
INTERPRO	Leucine-rich repeat	RT	4	8.1E-1	1.0E0	1.0E2
Annotation Cluster 104	Enrichment Score: 0.1		Count	P_Value	Benjamini	FDR
GOTERM_MF_FAT	voltage-gated channel activity	RT	4	7.5E-1	1.0E0	1.0E2
GOTERM_MF_FAT	voltage-gated ion channel activity	RT	4	7.5E-1	1.0E0	1.0E2
GOTERM_MF_FAT	voltage-gated cation channel activity	RT	3	7.9E-1	1.0E0	1.0E2
SP_PIR_KEYWORDS	voltage-gated channel	RT	3	8.0E-1	1.0E0	1.0E2
GOTERM_MF_FAT	alkali metal ion binding	RT	3	9.4E-1	1.0E0	1.0E2
Annotation Cluster 105	Enrichment Score: 0.07		Count	P_Value	Benjamini	FDR
SP_PIR_KEYWORDS	ionic channel	RT	6	7.5E-1	9.9E-1	1.0E2
GOTERM_MF_FAT	metal ion transmembrane transporter activity	RT	6	7.8E-1	1.0E0	1.0E2
GOTERM_MF_FAT	cation channel activity	RT	5	8.0E-1	1.0E0	1.0E2
GOTERM_MF_FAT	gated channel activity	RT	5	8.7E-1	1.0E0	1.0E2
GOTERM_MF_FAT	ion channel activity	RT	6	8.8E-1	1.0E0	1.0E2
GOTERM_MF_FAT	substrate specific channel activity	RT	6	9.0E-1	1.0E0	1.0E2
GOTERM_MF_FAT	channel activity	RT	6	9.1E-1	1.0E0	1.0E2
GOTERM_MF_FAT	passive transmembrane transporter activity	RT	6	9.2E-1	1.0E0	1.0E2
Annotation Cluster 106	Enrichment Score: 0.07		Count	P_Value	Benjamini	FDR
UP_SEQ_FEATURE	domain:KRAB	RT	6	7.9E-1	1.0E0	1.0E2
INTERPRO	Krueppel-associated box	RT	6	8.5E-1	1.0E0	1.0E2
SMART	KRAB	RT	6	9.1E-1	1.0E0	1.0E2
Annotation Cluster 107	Enrichment Score: 0.07		Count	P_Value	Benjamini	FDR
INTERPRO	EGF	RT	3	7.3E-1	1.0E0	1.0E2
INTERPRO	EGF-like, type 3	RT	4	7.6E-1	1.0E0	1.0E2
SP_PIR_KEYWORDS	egf-like domain	RT	4	8.3E-1	1.0E0	1.0E2
INTERPRO	EGF-like	RT	3	9.2E-1	1.0E0	1.0E2
INTERPRO	EGF-like region, conserved site	RT	4	9.4E-1	1.0E0	1.0E2
SMART	EGF	RT	3	9.5E-1	1.0E0	1.0E2
Annotation Cluster 108	Enrichment Score: 0.07		Count	P_Value	Benjamini	FDR
GOTERM_BP_FAT	nuclear division	RT	4	8.4E-1	1.0E0	1.0E2
GOTERM_BP_FAT	mitosis	RT	4	8.4E-1	1.0E0	1.0E2
GOTERM_BP_FAT	M phase of mitotic cell cycle	RT	4	8.5E-1	1.0E0	1.0E2
GOTERM_BP_FAT	organelle fission	RT	4	8.6E-1	1.0E0	1.0E2
SP_PIR_KEYWORDS	mitosis	RT	3	8.8E-1	1.0E0	1.0E2
Annotation Cluster 109	Enrichment Score: 0.06		Count	P_Value	Benjamini	FDR
UP_SEQ_FEATURE	zinc finger region:C2H2-type 8	RT	7	8.4E-1	1.0E0	1.0E2
UP_SEQ_FEATURE	zinc finger region:C2H2-type 6	RT	8	8.5E-1	1.0E0	1.0E2
UP_SEQ_FEATURE	zinc finger region:C2H2-type 9	RT	6	8.7E-1	1.0E0	1.0E2
UP_SEQ_FEATURE	zinc finger region:C2H2-type 7	RT	7	8.9E-1	1.0E0	1.0E2
Annotation Cluster 110	Enrichment Score: 0.06		Count	P_Value	Benjamini	FDR
GOTERM_BP_FAT	male gamete generation	RT	6	7.8E-1	1.0E0	1.0E2
GOTERM_BP_FAT	spermatogenesis	RT	6	7.8E-1	1.0E0	1.0E2
GOTERM_BP_FAT	gamete generation	RT	6	9.2E-1	1.0E0	1.0E2

Annotation Cluster 1	Enrichment Score: 6.89	G	Count	P_Value	Benjamini	FDR
GOTERM_BP_FAT	sexual reproduction	RT	7	9.2E-1	1.0E0	1.0E2
GOTERM_BP_FAT	multicellular organism reproduction	RT	7	9.4E-1	1.0E0	1.0E2
GOTERM_BP_FAT	reproductive process in a multicellular organism	RT	7	9.4E-1	1.0E0	1.0E2
Annotation Cluster 111	Enrichment Score: 0.05	G	Count	P_Value	Benjamini	FDR
GOTERM_BP_FAT	response to nutrient	RT	3	7.9E-1	1.0E0	1.0E2
GOTERM_BP_FAT	response to nutrient levels	RT	3	9.2E-1	1.0E0	1.0E2
GOTERM_BP_FAT	response to extracellular stimulus	RT	3	9.5E-1	1.0E0	1.0E2
Annotation Cluster 112	Enrichment Score: 0.05	G	Count	P_Value	Benjamini	FDR
GOTERM_BP_FAT	positive regulation of kinase activity	RT	4	8.7E-1	1.0E0	1.0E2
GOTERM_BP_FAT	positive regulation of transferase activity	RT	4	8.8E-1	1.0E0	1.0E2
GOTERM_BP_FAT	positive regulation of protein kinase activity	RT	3	9.5E-1	1.0E0	1.0E2
Annotation Cluster 113	Enrichment Score: 0.04	G	Count	P_Value	Benjamini	FDR
UP_SEQ_FEATURE	domain:PH	RT	4	8.5E-1	1.0E0	1.0E2
INTERPRO	Pleckstrin homology	RT	4	9.2E-1	1.0E0	1.0E2
SMART	PH	RT	4	9.5E-1	1.0E0	1.0E2
Annotation Cluster 114	Enrichment Score: 0.04	G	Count	P_Value	Benjamini	FDR
GOTERM_BP_FAT	visual perception	RT	4	8.3E-1	1.0E0	1.0E2
GOTERM_BP_FAT	sensory perception of light stimulus	RT	4	8.3E-1	1.0E0	1.0E2
SP_PIR_KEYWORDS	sensory transduction	RT	3	1.0E0	1.0E0	1.0E2
GOTERM_BP_FAT	sensory perception	RT	5	1.0E0	1.0E0	1.0E2
Annotation Cluster 115	Enrichment Score: 0.04	G	Count	P_Value	Benjamini	FDR
GOTERM_BP_FAT	positive regulation of protein metabolic process	RT	4	8.9E-1	1.0E0	1.0E2
GOTERM_BP_FAT	positive regulation of protein modification process	RT	3	9.1E-1	1.0E0	1.0E2
GOTERM_BP_FAT	positive regulation of cellular protein metabolic process	RT	3	9.6E-1	1.0E0	1.0E2
Annotation Cluster 116	Enrichment Score: 0.04	G	Count	P_Value	Benjamini	FDR
INTERPRO	Immunoglobulin	RT	4	7.8E-1	1.0E0	1.0E2
INTERPRO	Immunoglobulin subtype	RT	3	9.9E-1	1.0E0	1.0E2
SMART	IG	RT	3	1.0E0	1.0E0	1.0E2
Annotation Cluster 117	Enrichment Score: 0.03	G	Count	P_Value	Benjamini	FDR
SP_PIR_KEYWORDS	sh3 domain	RT	3	9.2E-1	1.0E0	1.0E2
INTERPRO	Src homology-3 domain	RT	3	9.3E-1	1.0E0	1.0E2
SMART	SH3	RT	3	9.6E-1	1.0E0	1.0E2
Annotation Cluster 118	Enrichment Score: 0	G	Count	P_Value	Benjamini	FDR
GOTERM_CC_FAT	intrinsic to membrane	RT	82	1.0E0	1.0E0	1.0E2
GOTERM_CC_FAT	integral to membrane	RT	77	1.0E0	1.0E0	1.0E2
SP_PIR_KEYWORDS	transmembrane	RT	71	1.0E0	1.0E0	1.0E2
UP_SEQ_FEATURE	transmembrane region	RT	70	1.0E0	1.0E0	1.0E2

609 terms were not clustered.

Please cite *Nature Protocols 2009; 4(1):44 & Genome Biology 2003; 4(5):P3* within any publication that makes use of any methods inspired by **DAVID**.

Term of Service | Contact Us | Site Map

National Institute of Allergy and Infectious Disease
The Database for Annotation, Visualization and Integrated Discovery

Gene Report

Help and Manual

44 record(s)

Download File

ID	GENE NAME	Related Genes	Species
ADAM metallopeptidase domain 19 (meltrin beta)	ADAM metallopeptidase domain 19 (meltrin beta)	RG	Homo sapiens
BRCA1 associated RING domain 1	BRCA1 associated RING domain 1	RG	Homo sapiens
Cas-Br-M (murine) ecotropic retroviral transforming sequence-like 1	Cas-Br-M (murine) ecotropic retroviral transforming sequence-like 1	RG	Homo sapiens
F-box and WD repeat domain containing 4	F-box and WD repeat domain containing 4	RG	Homo sapiens
F-box and leucine-rich repeat protein 3	F-box and leucine-rich repeat protein 3	RG	Homo sapiens
F-box protein 34	F-box protein 34	RG	Homo sapiens
F-box protein 38	F-box protein 38	RG	Homo sapiens
F-box protein 9	F-box protein 9	RG	Homo sapiens
LON peptidase N-terminal domain and ring finger 2	LON peptidase N-terminal domain and ring finger 2	RG	Homo sapiens
PEST proteolytic signal containing nuclear protein	PEST proteolytic signal containing nuclear protein	RG	Homo sapiens
RAD23 homolog A (S. cerevisiae)	RAD23 homolog A (S. cerevisiae)	RG	Homo sapiens
UBX domain protein 1	UBX domain protein 1	RG	Homo sapiens
ancient ubiquitous protein 1	ancient ubiquitous protein 1	RG	Homo sapiens
ankyrin repeat and SOCS box-containing 11	ankyrin repeat and SOCS box-containing 11	RG	Homo sapiens
ankyrin repeat and SOCS box-containing 15	ankyrin repeat and SOCS box-containing 15	RG	Homo sapiens
ankyrin repeat and SOCS box-containing 2	ankyrin repeat and SOCS box-containing 2	RG	Homo sapiens
ankyrin repeat and SOCS box-containing 3	ankyrin repeat and SOCS box-containing 3	RG	Homo sapiens
autocrine motility factor receptor	autocrine motility factor receptor	RG	Homo sapiens
calpain 3, (p94)	calpain 3, (p94)	RG	Homo sapiens
caspase 7, apoptosis-related cysteine peptidase	caspase 7, apoptosis-related cysteine peptidase	RG	Homo sapiens
cell division cycle 16 homolog (S. cerevisiae)	cell division cycle 16 homolog (S. cerevisiae)	RG	Homo sapiens
chromobox homolog 4 (Pc class homolog, Drosophila)	chromobox homolog 4 (Pc class homolog, Drosophila)	RG	Homo sapiens
complement factor H	complement factor H	RG	Homo sapiens
cytokine inducible SH2-containing protein	cytokine inducible SH2-containing protein	RG	Homo sapiens
fem-1 homolog b (C. elegans)	fem-1 homolog b (C. elegans)	RG	Homo sapiens
hect domain and RLD 2	hect domain and RLD 2	RG	Homo sapiens
lysine (K)-specific demethylase 2B	lysine (K)-specific demethylase 2B	RG	Homo sapiens
membrane-associated ring finger (C3HC4) 5	membrane-associated ring finger (C3HC4) 5	RG	Homo sapiens
membrane-associated ring finger (C3HC4) 7	membrane-associated ring finger (C3HC4) 7	RG	Homo sapiens
potassium channel modulatory factor 1	potassium channel modulatory factor 1	RG	Homo sapiens
ribonuclease H1	ribonuclease H1	RG	Homo sapiens
ring finger protein 216	ring finger protein 216	RG	Homo sapiens
seven in absentia homolog 1 (Drosophila)	seven in absentia homolog 1 (Drosophila)	RG	Homo sapiens
signal peptidase complex subunit 2 homolog (S. cerevisiae); signal peptidase complex subunit 2 homolog pseudogene	signal peptidase complex subunit 2 homolog (S. cerevisiae); signal peptidase complex subunit 2 homolog pseudogene	RG	Homo sapiens
similar to ribonucleic acid binding protein S1; RNA binding protein S1, serine-rich domain	similar to ribonucleic acid binding protein S1; RNA binding protein S1, serine-rich domain	RG	Homo sapiens
ubiquitin D	ubiquitin D	RG	Homo sapiens
ubiquitin protein ligase E3 component n-recognin 3 (putative)	ubiquitin protein ligase E3 component n-recognin 3 (putative)	RG	Homo sapiens
ubiquitin specific peptidase 24	ubiquitin specific peptidase 24	RG	Homo sapiens
ubiquitin specific peptidase 49	ubiquitin specific peptidase 49	RG	Homo sapiens

ID	GENE NAME	Related Genes	Species
ubiquitin specific peptidase 53	ubiquitin specific peptidase 53	RG	Homo sapiens
ubiquitin-conjugating enzyme E2 variant 1; ubiquitin-conjugating enzyme E2 variant 1 pseudogene 2; transmembrane protein 189; TMEM189-UBE2V1 readthrough transcript	ubiquitin-conjugating enzyme E2 variant 1; ubiquitin-conjugating enzyme E2 variant 1 pseudogene 2; transmembrane protein 189; TMEM189-UBE2V1 readthrough transcript	RG	Homo sapiens
ubiquitin-conjugating enzyme E2G 1 (UBC7 homolog, yeast)	ubiquitin-conjugating enzyme E2G 1 (UBC7 homolog, yeast)	RG	Homo sapiens
ubiquitin-conjugating enzyme E2L 6	ubiquitin-conjugating enzyme E2L 6	RG	Homo sapiens
v-rel reticuloendotheliosis viral oncogene homolog A (avian)	v-rel reticuloendotheliosis viral oncogene homolog A (avian)	RG	Homo sapiens

Please cite *Nature Protocols 2009; 4(1):44 & Genome Biology 2003; 4(5):P3* within any publication that makes use of any methods inspired by **DAVID**.

Term of Service | Contact Us | Site Map

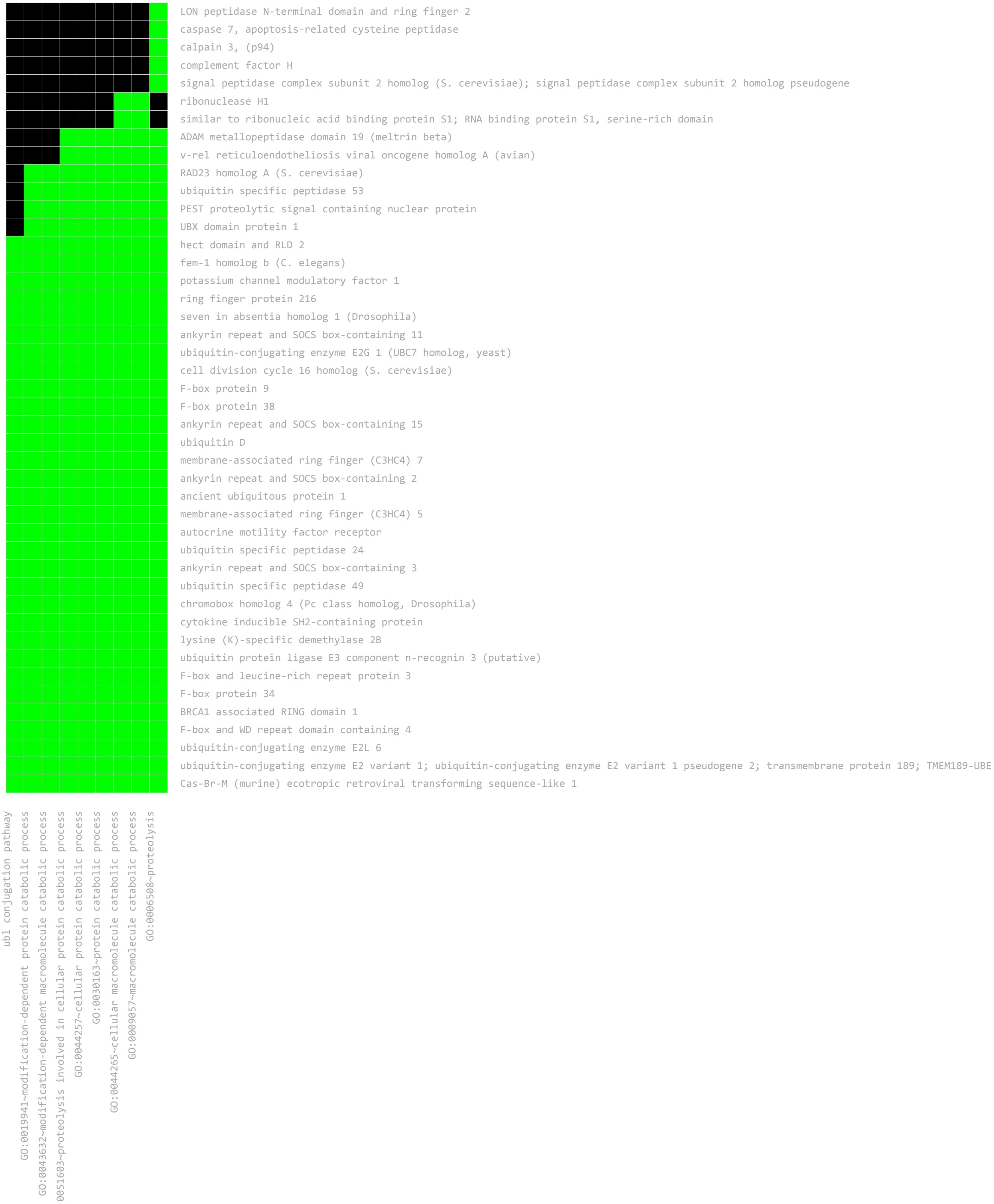
LON peptidase N-terminal domain and ring finger 2
caspase 7, apoptosis-related cysteine peptidase
calpain 3, (p94)
complement factor H
signal peptidase complex subunit 2 homolog (S. cerevisiae); signal peptidase complex subunit 2 homolog pseudogene
ribonuclease H1
similar to ribonucleic acid binding protein S1; RNA binding protein S1, serine-rich domain
ADAM metallopeptidase domain 19 (meltrin beta)
v-rel reticuloendotheliosis viral oncogene homolog A (avian)
RAD23 homolog A (S. cerevisiae)
ubiquitin specific peptidase 53
PEST proteolytic signal containing nuclear protein
UBX domain protein 1
hect domain and RLD 2
fem-1 homolog b (C. elegans)
potassium channel modulatory factor 1
ring finger protein 216
seven in absentia homolog 1 (Drosophila)
ankyrin repeat and SOCS box-containing 11
ubiquitin-conjugating enzyme E2G 1 (UBC7 homolog, yeast)
cell division cycle 16 homolog (S. cerevisiae)
F-box protein 9
F-box protein 38
ankyrin repeat and SOCS box-containing 15
ubiquitin D
membrane-associated ring finger (C3HC4) 7
ankyrin repeat and SOCS box-containing 2
ancient ubiquitous protein 1
membrane-associated ring finger (C3HC4) 5
autocrine motility factor receptor
ubiquitin specific peptidase 24
ankyrin repeat and SOCS box-containing 3
ubiquitin specific peptidase 49
chromobox homolog 4 (Pc class homolog, Drosophila)
cytokine inducible SH2-containing protein
lysine (K)-specific demethylase 2B
ubiquitin protein ligase E3 component n-recognin 3 (putative)
F-box and leucine-rich repeat protein 3
F-box protein 34
BRCA1 associated RING domain 1
F-box and WD repeat domain containing 4
ubiquitin-conjugating enzyme E2L 6
ubiquitin-conjugating enzyme E2 variant 1; ubiquitin-conjugating enzyme E2 variant 1 pseudogene 2; transmembrane protein 189; TMEM189-UBE
Cas-Br-M (murine) ecotropic retroviral transforming sequence-like 1
ubl conjugation pathway
GO:0019941~modification-dependent protein catabolic process
GO:0043632~modification-dependent macromolecule catabolic process
GO:0051603~proteolysis involved in cellular protein catabolic process
GO:0044257~cellular protein catabolic process
GO:0030163~protein catabolic process
GO:0044265~cellular macromolecule catabolic process
GO:0009057~macromolecule catabolic process
GO:0006508~proteolysis

National Institute of Allergy and Infectious Disease
The Database for Annotation, Visualization and Integrated Discovery

Gene Report

Help and Manual

Current Gene List: List_2
Current Background: Homo sapiens
391 DAVID IDs
14 record(s)

Download File

ILLUMINA_ID	GENE NAME	Related Genes	Species
ILMN_1676241	BCL6 co-repressor	RG	Homo sapiens
ILMN_2074258	BRCA1 associated RING domain 1	RG	Homo sapiens
ILMN_2073732	Cas-Br-M (murine) ecotropic retroviral transforming sequence-like 1	RG	Homo sapiens
ILMN_1794187	F-box and leucine-rich repeat protein 3	RG	Homo sapiens
ILMN_1717063	F-box protein 9	RG	Homo sapiens
ILMN_1666258	autocrine motility factor receptor	RG	Homo sapiens
ILMN_1744239	fem-1 homolog b (C. elegans)	RG	Homo sapiens
ILMN_1813475	hect domain and RLD 2	RG	Homo sapiens
ILMN_2380566	seven in absentia homolog 1 (Drosophila)	RG	Homo sapiens
ILMN_2197659	solute carrier family 14 (urea transporter), member 1 (Kidd blood group)	RG	Homo sapiens
ILMN_2171640	ubiquitin protein ligase E3 component n-recognin 3 (putative)	RG	Homo sapiens
ILMN_2368713	ubiquitin-conjugating enzyme E2 variant 1; ubiquitin-conjugating enzyme E2 variant 1 pseudogene 2; transmembrane protein 189; TMEM189-UBE2V1 readthrough transcript	RG	Homo sapiens
ILMN_1814465	ubiquitin-conjugating enzyme E2G 1 (UBC7 homolog, yeast)	RG	Homo sapiens
ILMN_1769520	ubiquitin-conjugating enzyme E2L 6	RG	Homo sapiens

Please cite *Nature Protocols 2009; 4(1):44 & Genome Biology 2003; 4(5):P3* within any publication that makes use of any methods inspired by **DAVID**.

Term of Service | Contact Us | Site Map

DAVID Bioinformatics Resources 6.7
National Institute of Allergy and Infectious Diseases (NIAID), NIH

***** Announcing DAVID 6.8 Beta with updated Knowledgebase (more info). You may explore the new version at david-d.ncifcrf.gov. *** DAVID 6.8 w
6.7 will be available on our development site until at least January 15, 2017.**

2D View

Help

☐ corresponding gene-term association positively reported ☐ corresponding gene-term association not reported yet

Download File

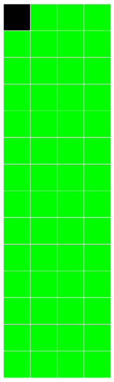

ubiquitin-conjugating enzyme E2 variant 1; ubiquitin-conjugating enzyme E2 variant 1 pseudogene 2; transmembrane protein 189; TMEM189-UBE2V1 readthrough
fem-1 homolog b (C. elegans)
hect domain and RLD 2
Cas-Br-M (murine) ecotropic retroviral transforming sequence-like 1
autocrine motility factor receptor
seven in absentia homolog 1 (Drosophila)
ubiquitin-conjugating enzyme E2G 1 (UBC7 homolog, yeast)
ubiquitin protein ligase E3 component n-recognin 3 (putative)
F-box protein 9
F-box and leucine-rich repeat protein 3
BRCA1 associated RING domain 1
BCL6 co-repressor
solute carrier family 14 (urea transporter), member 1 (Kidd blood group)
ubiquitin-conjugating enzyme E2L 6

GO:0004842~ubiquitin-protein ligase activity
GO:0019787~small conjugating protein ligase activity
GO:0016881~acid-amino acid ligase activity
GO:0016879~ligase activity, forming carbon-nitrogen bonds

Gene Report

Help and Manual

Current Gene List: List_2
Current Background: Homo sapiens
391 DAVID IDs
56 record(s)

Download File

ILLUMINA_ID	GENE NAME	Related Genes	Species
ILMN_1741572	A kinase (PRKA) anchor protein 8	RG	Homo sapiens
ILMN_1797341	AT rich interactive domain 1A (SWI-like)	RG	Homo sapiens
ILMN_2278235	C-terminal binding protein 1	RG	Homo sapiens
ILMN_1746257	DAZ associated protein 1	RG	Homo sapiens
ILMN_1735461	DEAD (Asp-Glu-Ala-Asp) box polypeptide 21	RG	Homo sapiens
ILMN_1795218	DEAH (Asp-Glu-Ala-His) box polypeptide 30	RG	Homo sapiens
ILMN_1761828	E2F transcription factor 4, p107/p130-binding	RG	Homo sapiens
ILMN_1768127	EBNA1 binding protein 2	RG	Homo sapiens
ILMN_1674024	IKAROS family zinc finger 5 (Pegasus)	RG	Homo sapiens
ILMN_1709483	MRE11 meiotic recombination 11 homolog A (S. cerevisiae)	RG	Homo sapiens
ILMN_2322986	MYC induced nuclear antigen	RG	Homo sapiens
ILMN_2363027	RAD51 homolog (RecA homolog, E. coli) (S. cerevisiae)	RG	Homo sapiens
ILMN_2158336	SH3-domain GRB2-like endophilin B2	RG	Homo sapiens
ILMN_1678729	SIL1 homolog, endoplasmic reticulum chaperone (S. cerevisiae)	RG	Homo sapiens
ILMN_1703427	SON DNA binding protein	RG	Homo sapiens
ILMN_1737535	TAF4 RNA polymerase II, TATA box binding protein (TBP)-associated factor, 135kDa	RG	Homo sapiens
ILMN_1697420	TERF1 (TRF1)-interacting nuclear factor 2	RG	Homo sapiens
ILMN_1711566	TIMP metallopeptidase inhibitor 1	RG	Homo sapiens
ILMN_2130838	UTP11-like, U3 small nucleolar ribonucleoprotein, (yeast)	RG	Homo sapiens
ILMN_2105983	X-ray repair complementing defective repair in Chinese hamster cells 5 (double-strand-break rejoining)	RG	Homo sapiens
ILMN_1670878	YTH domain containing 1	RG	Homo sapiens
ILMN_1770892	YY1 transcription factor	RG	Homo sapiens
ILMN_1679797	adenosine deaminase, RNA-specific, B1 (RED1 homolog rat)	RG	Homo sapiens
ILMN_1666385	calmodulin 3 (phosphorylase kinase, delta); calmodulin 2 (phosphorylase kinase, delta); calmodulin 1 (phosphorylase kinase, delta)	RG	Homo sapiens
ILMN_2362681	carboxylesterase 2 (intestine, liver)	RG	Homo sapiens
ILMN_2362974	caspase 7, apoptosis-related cysteine peptidase	RG	Homo sapiens
ILMN_2256765	cell division cycle 16 homolog (S. cerevisiae)	RG	Homo sapiens
ILMN_1692390	cyclin T1	RG	Homo sapiens
ILMN_2313074	death effector domain containing	RG	Homo sapiens
ILMN_1660663, ILMN_1664560	dual-specificity tyrosine-(Y)-phosphorylation regulated kinase 1A	RG	Homo sapiens
ILMN_2312719	exosome component 9	RG	Homo sapiens
ILMN_2188374	exportin, tRNA (nuclear export receptor for tRNAs); similar to Exportin-T (tRNA exportin) (Exportin(tRNA))	RG	Homo sapiens
ILMN_1761113	guanine nucleotide binding protein-like 2 (nucleolar)	RG	Homo sapiens
ILMN_2324056	guanine nucleotide binding protein-like 3 (nucleolar)	RG	Homo sapiens
ILMN_2220283	heterogeneous nuclear ribonucleoprotein A1-like 3; similar to heterogeneous nuclear ribonucleoprotein A1; heterogeneous nuclear ribonucleoprotein A1 pseudogene 2; heterogeneous nuclear ribonucleoprotein A1; heterogeneous nuclear ribonucleoprotein A1 pseudogene	RG	Homo sapiens
ILMN_2231242	high-mobility group box 1; high-mobility group box 1-like 10	RG	Homo sapiens
ILMN_1772455	histone deacetylase 3	RG	Homo sapiens
ILMN_1728521	histone deacetylase 7	RG	Homo sapiens
ILMN_2317463	integrator complex subunit 1	RG	Homo sapiens
ILMN_1679483	integrator complex subunit 10	RG	Homo sapiens
ILMN_1725169	integrator complex subunit 12	RG	Homo sapiens
ILMN_1794260	lysine (K)-specific demethylase 2B	RG	Homo sapiens
ILMN_1706539	lysine (K)-specific demethylase 3B	RG	Homo sapiens
ILMN_2389347	nuclear receptor subfamily 3, group C, member 1 (glucocorticoid receptor)	RG	Homo sapiens
ILMN_1771835	nucleoporin 54kDa	RG	Homo sapiens
ILMN_1710844	poly (ADP-ribose) polymerase family, member 10	RG	Homo sapiens
ILMN_1659411	polymerase (RNA) II (DNA directed) polypeptide C, 33kDa	RG	Homo sapiens
ILMN_1719303	prolyl 4-hydroxylase, beta polypeptide	RG	Homo sapiens
ILMN_1668778	protein kinase, AMP-activated, alpha 2 catalytic subunit	RG	Homo sapiens
ILMN_1701855	protein phosphatase 1, catalytic subunit, gamma isoform	RG	Homo sapiens
ILMN_1761175	ribosomal protein S6 kinase, 70kDa, polypeptide 2	RG	Homo sapiens

ILLUMINA_ID	GENE NAME	Related Genes	Species
ILMN_2375386	similar to ribonucleic acid binding protein S1; RNA binding protein S1, serine-rich domain	RG	Homo sapiens
ILMN_2175075	splicing factor, arginine/serine-rich 4	RG	Homo sapiens
ILMN_1660368	transformation/transcription domain-associated protein	RG	Homo sapiens
ILMN_1705266	v-rel reticuloendotheliosis viral oncogene homolog A (avian)	RG	Homo sapiens
ILMN_1661636	zinc finger, MYM-type 2	RG	Homo sapiens

Please cite *Nature Protocols 2009; 4(1):44 & Genome Biology 2003; 4(5):P3* within any publication that makes use of any methods inspired by **DAVID**.

Term of Service | Contact Us | Site Map

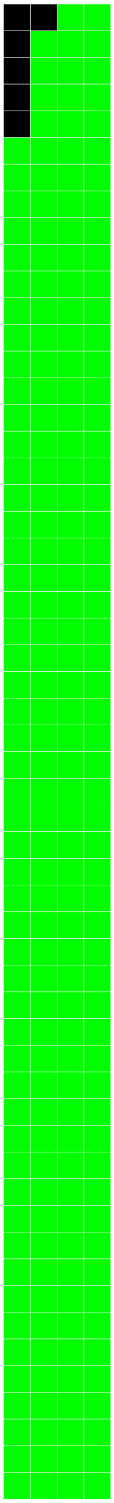

TIMP metallopeptidase inhibitor 1
DEAH (Asp-Glu-Ala-His) box polypeptide 30
carboxylesterase 2 (intestine, liver)
prolyl 4-hydroxylase, beta polypeptide
SIL1 homolog, endoplasmic reticulum chaperone (S. cerevisiae)
RAD51 homolog (RecA homolog, E. coli) (S. cerevisiae)
MRE11 meiotic recombination 11 homolog A (S. cerevisiae)
C-terminal binding protein 1
IKAROS family zinc finger 5 (Pegasus)
guanine nucleotide binding protein-like 2 (nucleolar)
transformation/transcription domain-associated protein
nuclear receptor subfamily 3, group C, member 1 (glucocorticoid receptor)
YY1 transcription factor
histone deacetylase 3
protein phosphatase 1, catalytic subunit, gamma isoform
exportin, tRNA (nuclear export receptor for tRNAs); similar to Exportin-T (tRNA exportin) (Exportin(tRNA))
nucleoporin 54kDa
lysine (K)-specific demethylase 3B
DEAD (Asp-Glu-Ala-Asp) box polypeptide 21
AT rich interactive domain 1A (SWI-like)
caspase 7, apoptosis-related cysteine peptidase
SON DNA binding protein
polymerase (RNA) II (DNA directed) polypeptide C, 33kDa
high-mobility group box 1; high-mobility group box 1-like 10
v-rel reticuloendotheliosis viral oncogene homolog A (avian)
histone deacetylase 7
integrator complex subunit 12
E2F transcription factor 4, p107/p130-binding
splicing factor, arginine/serine-rich 4
integrator complex subunit 10
ribosomal protein S6 kinase, 70kDa, polypeptide 2
cyclin T1
calmodulin 3 (phosphorylase kinase, delta); calmodulin 2 (phosphorylase kinase, delta); calmodulin 1 (phosphorylase kinase, delta)
A kinase (PRKA) anchor protein 8
adenosine deaminase, RNA-specific, B1 (RED1 homolog rat)
TERF1 (TRF1)-interacting nuclear factor 2
EBNA1 binding protein 2
exosome component 9
cell division cycle 16 homolog (S. cerevisiae)
UTP11-like, U3 small nucleolar ribonucleoprotein, (yeast)
similar to ribonucleic acid binding protein S1; RNA binding protein S1, serine-rich domain
YTH domain containing 1
TAF4 RNA polymerase II, TATA box binding protein (TBP)-associated factor, 135kDa
heterogeneous nuclear ribonucleoprotein A1-like 3; similar to heterogeneous nuclear ribonucleoprotein A1; heterogeneous nuclear ribonucleoprotein A1 pse
integrator complex subunit 1
poly (ADP-ribose) polymerase family, member 10
DAZ associated protein 1
X-ray repair complementing defective repair in Chinese hamster cells 5 (double-strand-break rejoining)
MYC induced nuclear antigen
lysine (K)-specific demethylase 2B
dual-specificity tyrosine-(Y)-phosphorylation regulated kinase 1A
death effector domain containing
guanine nucleotide binding protein-like 3 (nucleolar)
zinc finger, MYM-type 2
protein kinase, AMP-activated, alpha 2 catalytic subunit
SH3-domain GRB2-like endophilin B2

GO:0031981~nuclear lumen
GO:0070013~intracellular organelle lumen
GO:0043233~organelle lumen
GO:0031974~membrane-enclosed lumen
