# Supplementary figures and images for: Patients experiencing statin-induced myalgia exhibit a unique program of skeletal muscle gene expression following statin re-challenge
Source: PLoS One. 2017 Aug 3;12(8):e0181308. doi: 10.1371/journal.pone.0181308 (PMC5542661; doi:10.1371/journal.pone.0181308)

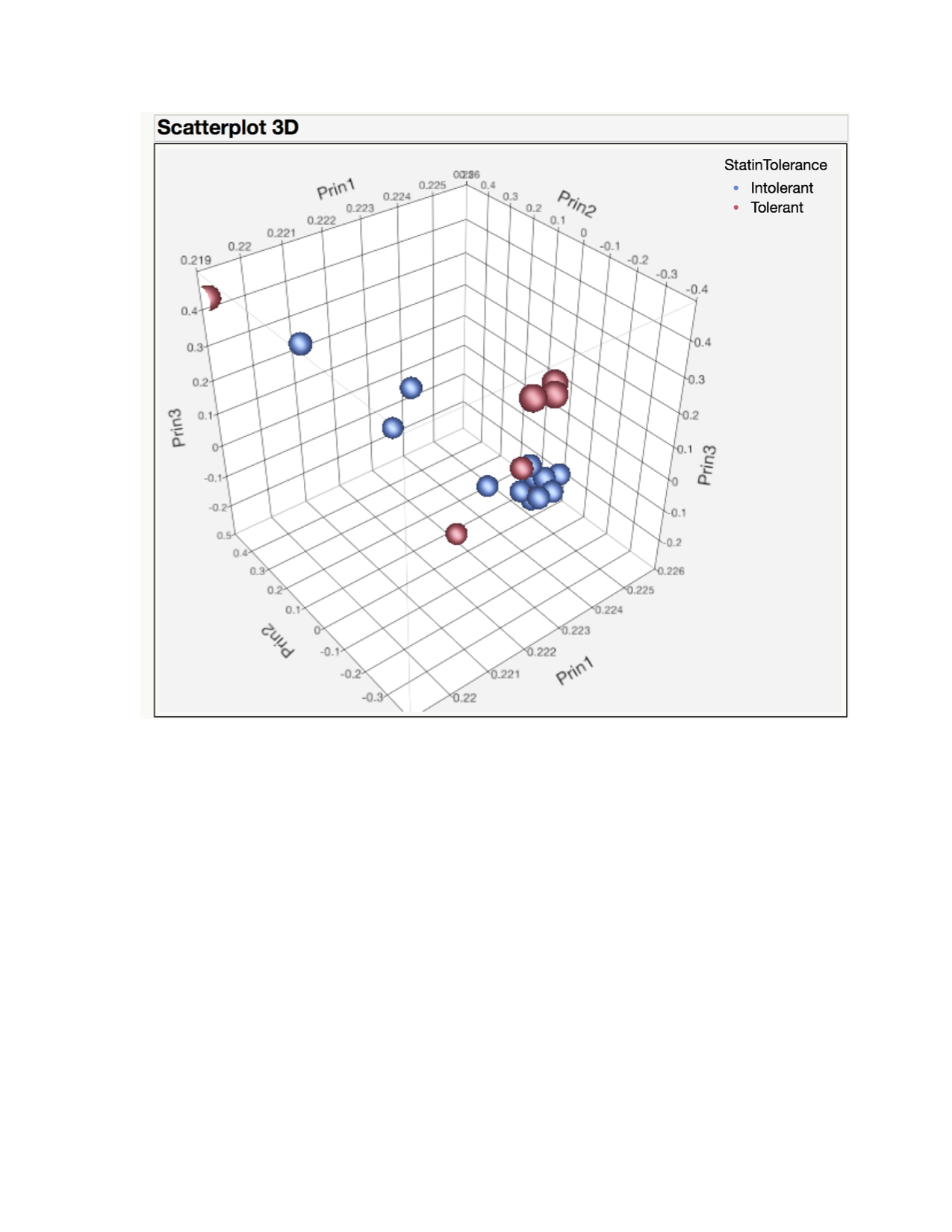

Supplement: S1 Fig — Initial PCA analysis of 23 samples identified 3 outliers that were removed from fruther analysis. PC analysis of the remaining 20 samples (shown here) showed that PC1 explained the highest amount of variance. In bivariate analysis Vitamin D level was moderately correlated with PC1 and PC3. Batch (i.e. the array slide on which a sample was loaded was signficantly associated with PC2 and PC3. Statin tolerance status was significantly associated with PC3. Based upon the results of this analysis linear regression analysis was performed to evaluate the association between statin intolerance and gene expression with adjustment for vitamin D levels and batch effect. (TIF) [file pone.0181308.s004.tif]

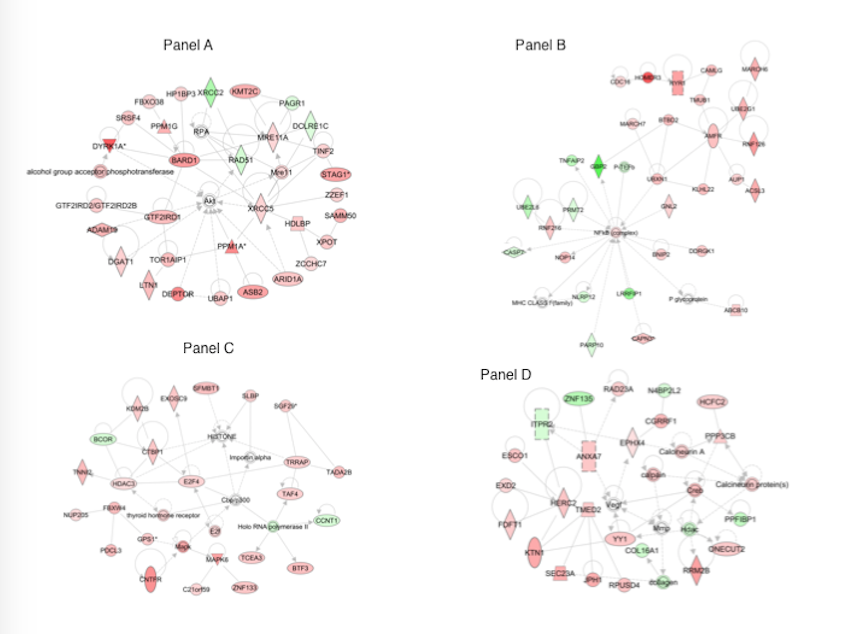

Supplement: S2 Fig — Genes exhibiting differential expression in skeletal muscle of statin intolerant vs tolerant study participants are denoted in green (lower expression in statin intolerant) and pink/red (higher expression in statin intolerant study subjects). Regulatory molecules shared by genes within each network some of which are DEGs and some are not are identified as "hubs". Solid lines denote positive interaction and dashed lines inhibitory influences. Genes denoted by circles represent other proteins, triangles represent kinases, inverted triangles represent phosphatases, and diamonds represent enzymes. Gene networks identified are (Panel A): Network 1, Cell Cycle/Cancer (Panel B): Network 2, Cell Cycle/Small Molecule Biochemistry (Panel C): Network 3, Gene Expression/Cellular Assembly and Organization (Panel D): Network 5, Skeletal and Muscular Development and Function. (TIF) [file pone.0181308.s005.tif]

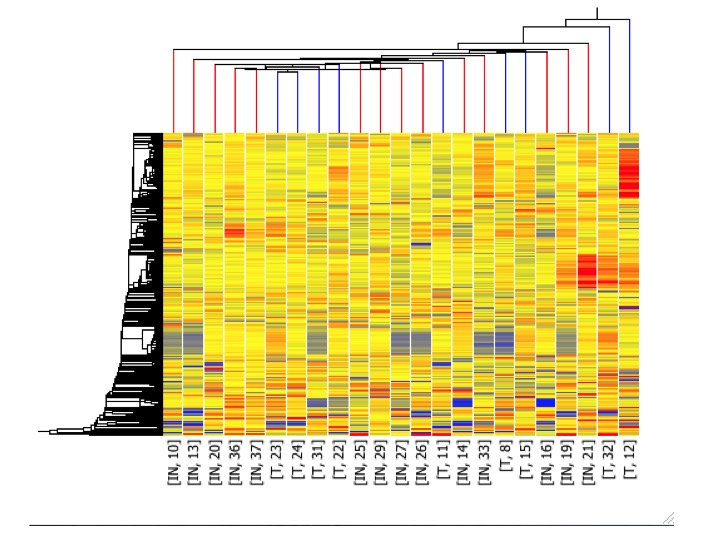

Supplement: S3 Fig — Hierarchical analysis shows no clear differentiation in gene expression in PBMCs isolated from statin tolerant and intolerant subjects. (TIF) [file pone.0181308.s006.tif]
